# Oviposition stimulants underlying different preferences between host races in the leaf-mining moth *Acrocercops transecta* (Lepidoptera: Gracillariidae)

**DOI:** 10.1038/s41598-022-18238-0

**Published:** 2022-08-25

**Authors:** Tomoko Katte, Shota Shimoda, Takuya Kobayashi, Ayako Wada-Katsumata, Ritsuo Nishida, Issei Ohshima, Hajime Ono

**Affiliations:** 1 Graduate School of Agriculture, Kyoto University, Kyoto, 606-8502 Japan; 2 Department of Entomology and Plant Pathology, and W.M. Keck Center for Behavioral Biology, North Carolina State University, Raleigh, NC 27695-7613 USA; 3 Department of Life and Environmental Sciences, Kyoto Prefectural University, Kyoto, 606-8522 Japan; 4 Center for Frontier Natural History, Kyoto Prefectural University, Kyoto, 606-8522 Japan

**Keywords:** Chemical ecology, Coevolution

## Abstract

The importance of plant chemistry in the host specialization of phytophagous insects has been emphasized. However, only a few chemicals associated with host shifting have been characterized. Herein, we focus on the leaf-mining moth *Acrocercops transecta* (Gracillariidae) consisting of ancestral *Juglans* (Juglandaceae)- and derived *Lyonia* (Ericaceae)-associated host races. The females of the *Lyonia* race laid eggs on a cover glass treated with an *L*. *ovalifolia* leaf extract; the extract was fractionated using silica gel and ODS column chromatography to isolate the oviposition stimulants. From a separated fraction, two analogous *Lyonia*-specific triterpenoid glycosides were characterized as oviposition stimulants. Furthermore, we observed probable contact chemosensilla on the distal portion of the female antennae. *Lyonia* race females laid their eggs on the non-host *Juglans* after the leaves were treated with a *Lyonia*-specific oviposition stimulant, although they do not lay eggs on *Juglans*. These results suggest that *Lyonia* race females do not lay eggs on *Juglans* leaves because the leaves do not contain specific oviposition stimulant(s). Otherwise, the activity of the oviposition stimulants overcomes oviposition deterrents contained in *Juglans* leaves. This paper describes the roles of plant chemicals in the different preferences between host races associated with distantly related plant taxa.

Most phytophagous insects specialize in specific plant taxa (e.g., families or genera)^1,2^. Host specialization plays a key role in the extreme diversification of phytophagous insects because various traits have been developed to optimally adapt to a subset of host plants^3,4^. One important factor constraining the host range of insect specialists is plant chemistry^1^. In the process of adapting to novel plants, herbivores must overcome the defensive substances contained within the plants and develop a recognition system that would identify specific plants as suitable hosts. The adaptation by herbivores to the defenses of the novel host plants conversely promotes the evolution of defensive traits in the plants. Thus, a co-evolutionary process associated with defensive compounds could strongly drive diversification in both insect herbivores and plants^5–8^. In this process, chemosensory cues recognized by insect herbivores play an important role in the selection of suitable and/or non-hazardous host plants^2^.

Host shifts and subsequent host race/ecotype formations have been considered as an important initial step of ecological speciation in phytophagous insects^9,10^. Host races in phytophagous insects are sympatrically distributed but genetically differentiated populations that utilize different host plant species^9^. In butterfly-plant interactions, it has been proposed that host shifts occur more frequently between closely related plants than between distantly related plants. This is thought to be because related plant taxa often contain similar chemical compounds that can be readily overcome by insect herbivores that have shifted from a related species^11,12^. Phylogenetic relationships among monophagous leaf beetles of the genus *Blepharida* are associated with the defensive compounds in plants within the genus *Bursera*, rather than the molecular phylogeny and geographic availability, suggesting that host plant chemistry is important for host shifting of phytophagous insects within closely related plant species^8,13^. Rapid host race formation in the apple maggot fly *Rhagoletis pomonella*, from its native host hawthorn to apples introduced later as their new host, has been proposed as the initial stage of sympatric speciation^14,15^. In this instance, chemical factors, that is, fruit odors contained in the host and non-host fruits, are intimately involved in sympatric host shifting^16–19^. Notably, host race formation between distantly related plant groups has also been observed^20,21^. For host selection, the oviposition preferences of the females determine the fate of their offspring, because the hatched larvae mostly do not have ability to move away far from the leaves onto which they have been deposited. Since the chemosensory cues in a host or non-host plant play a major role in host acceptance or rejection by insects^22^, the recognition of chemical cues contained within a derived host plant has special significance in host shifting^23^. Therefore, the presence of common or similar secondary metabolites, even between distantly related plants, could promote host shifting as recognition cues for ovipositing females^24^. Contrarily, insect herbivores may acquire a new capability to detect novel compound(s) contained within a new host plant, which would enable the insects to shift to distantly related plant species. Here, one question arises: how do newly colonized populations lose the recognition for their original hosts after the completion of shifting? One possibility is that they detect any chemicals contained in the original host as a deterrent after host shifts occur. Another possibility is that they lose the ability to detect the compounds contained within their original hosts. However, the chemosensory cues, especially contact cues, involved in host shifting have been rarely characterized. Therefore, we focused on how changes in the detection of contact chemosensory cues can be linked to host shifting in phytophagous insects. In this regard, we initially characterized bioactive compounds associated with intraspecific host shifting.

Herein, we focused on host races, namely *Juglans* and *Lyonia* races, in a leaf-mining micro-moth, *Acrocercops transecta* (Gracillariidae), that utilizes distantly related plant species: either juglandaceous species (Juglandaceae) or *Lyonia ovalifolia* (Ericaceae), which respectively belong to Rosids or Asterids^25^. The two host races exhibit different female oviposition preferences and larval performances^26^. When given a choice between a host or non-host plant, gravid females of each host race oviposit only on their own hosts: females of *Juglans* and *Lyonia* races exclusively laid eggs on *Juglans* and *Lyonia* leaves, respectively. Regardless of the distinct host preferences in adult females, larvae of the derived race, *Lyonia* race, can survive and grow on juglandaceous plants into adulthood, although larvae of the *Juglans* race cannot survive on the derived host, *Lyonia*^20,26^. Therefore, the toxic effects (and/or hindered digestion) of plant secondary metabolites could cause the non-viability of the *Juglans* race on *Lyonia*^26^. Phylogenetic studies using mitochondrial DNA sequences have suggested that the *Juglans* race is ancestral in *A*. *transecta*^20,26^. These results suggest that the *Lyonia* race evolved from the *Juglans* race via physiological adaptation to *Lyonia ovalifolia*. Since the two host races are sympatric at many sites in the wild^25^, adaptation to *Lyonia* could promote host shifting and play an important role in the maintenance of the two host races^27^.

As chemosensory cues in the plants play a major role in host acceptance or rejection by insects^22,23,28^, we predict that the detection of chemicals in a new plant has special significance in host shifting. Orientation to and landing on a plant are mainly guided by volatile chemicals primarily detected by the olfactory sensilla located on the antennae^29^. The final decision to accept or reject a particular plant depends on the contact chemosensory cues. The contact chemosensory organs that recognize host plants have been well characterized in lepidopteran species, particularly in butterflies^30–33^. While foreleg tarsi are responsible for the recognition of oviposition stimulants in most cases, antennal contact chemosensilla may also be responsible for the detection of oviposition stimulants in the monarch butterfly *Danaus plexippus*^33^. While the cuticle overlying the olfactory sensilla has numerous small pores that permit the entry of volatile chemicals, contact chemosensilla are characterized by a single pore at the tip that allows direct contact with passing fluids and a socket structure at the base^34^. To elicit oviposition behavior, a specific combination of chemicals, including highly polar compounds, is required for several species in the family Papilionidae^35–37^.

To understand the process of host shifting, as mediated by chemical factors, we addressed the following issues: (1) How do *A*. *transecta* females recognize their host plants? Which chemosensory organ(s) is/are necessary to detect host leaf chemicals, and what type of chemosensilla is/are involved in the system? (2) Which chemicals contained within *L. ovalifolia* leaves are responsible for host recognition by *Lyonia* race females? Are the chemicals specific to *Lyonia* leaves or shared by both *Lyonia* and *Juglans* leaves? (3) Why do females of the *Lyonia* race not oviposit on *Juglans* leaves? Do *Juglans* leaves lack specific oviposition stimulants or do the leaves contain oviposition deterrents? Herein, we describe the roles of plant chemicals associated with host shifting between distantly related plant taxa.

## Results

### Host plant recognition by gravid females via chemosensory organs

**Figure 1:**
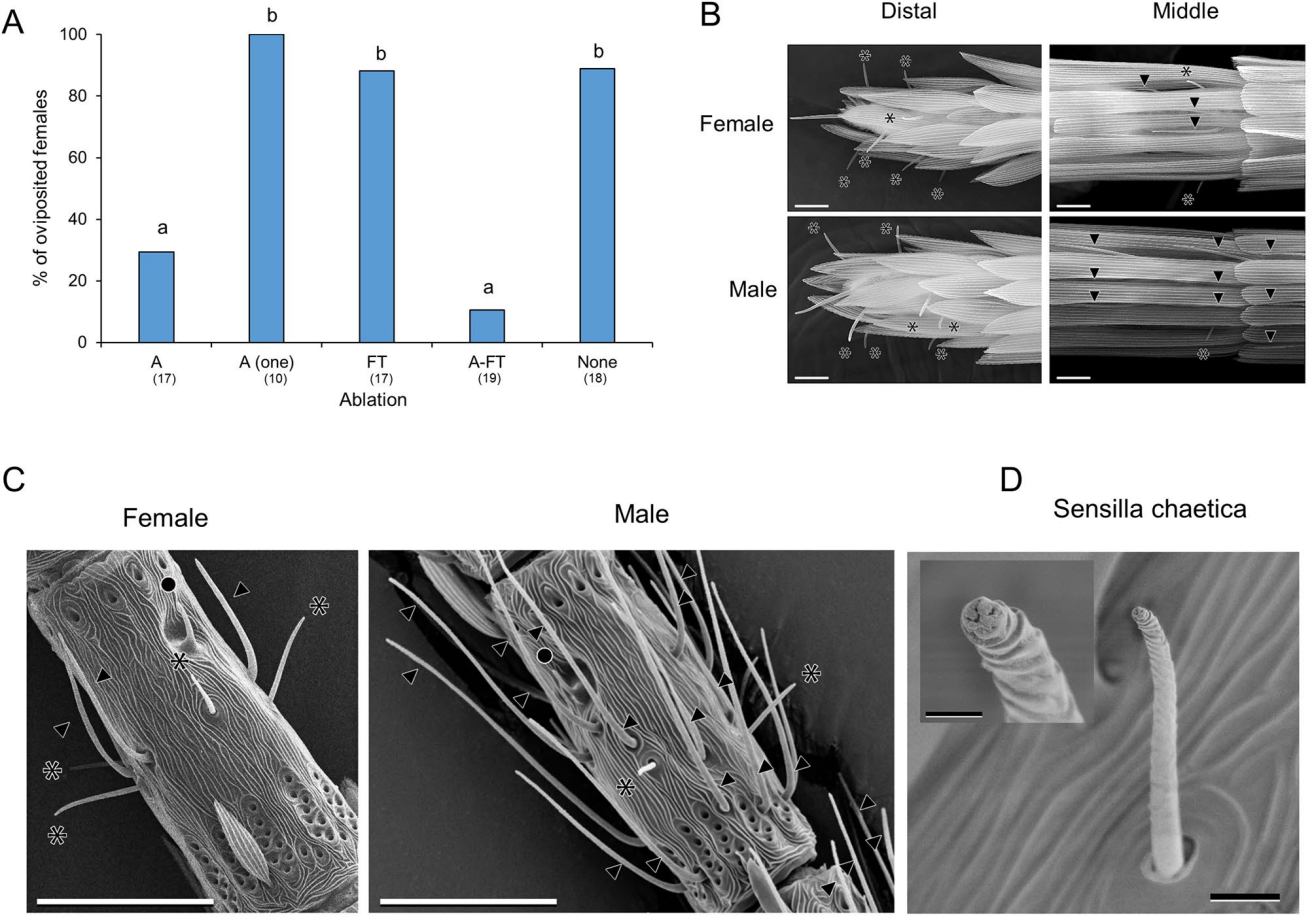
Host recognition via chemosensory organs. (**A**) Oviposition responses of *Lyonia* race females after ablating the following chemosensory organs, A: antennae; A (one): one of pair of antennae; FT: foreleg tarsi; A-FT: antennae and foreleg tarsi; None: untreated. Different letters indicate statistically significant differences among the treatments by Fisher’s exact test, *p* < 0.01. Numbers in parentheses represent the number of tested animals. (**B**) SEM images of distal and middle portion of female and male antennae. Scale bars: 10 μm. (**C**) Middle portion of antennae without scales. Scale bars: 25 μm. (**D**) Sensilla chaetica of female. Scale bar: 2 μm for whole image of sensillum and 0.5 μm for the image of tip. Symbols indicated sensilla chaetica (asterisk), sensilla basiconica (filled circle) and sensilla trichodea (filled arrowhead).

We observed that females of *A*. *transecta* tap leaves using their antennae during the search for host plants (electronic supplementary material, movie S1). To evaluate how chemosensory systems are involved in host recognition by the *Lyonia* race, we surgically removed the candidate chemosensory organs, that is, the antennae or foreleg tarsi, or both, of the gravid females. Ablation of the antennae resulted in a marked reduction in oviposition, but this treatment did not completely prevent oviposition (Fig. 1A, Table S1). When one of a pair of antennae was ablated, all the tested females laid eggs. When both antennae and foreleg tarsi were ablated, the females rarely oviposited; however, 2 out of 19 females did oviposit. Since there was no significant difference in the oviposition responses between antennae-ablated and both antennae- and tarsi-ablated females, the antenna may be considered a primary chemosensory organ that recognizes host plants.

We then observed the middle and distal portions of the antennae of both females and males. According to the general morphological classification method of insect sensory receptor organs^34^, at least three types of chemosensory-hair-like structures were found in both females and males:

(i) Sensilla chaetica protruding between the scales (Fig. 1B and C, asterisk) and approximately 10–15 μm in length are observed. The sensilla have characteristic straight hairs comprising a socket at the base and a pore on the blunt tip (Fig. 1D), suggesting that they are contact chemosensillum in insects^34^.
(ii) Sensilla basiconica under the scales (Fig. 1C, filled circle) and approximately 5–10 μm in length are observed. The sensilla have a blunt curved shape and a corrugated surface. Additionally, the sensilla have multiple slit structures on the surface and lack a socket at the base, suggesting that they are olfactory sensilla.
(iii) Sensilla trichodea, including a few subtypes under the scales (Fig. 1B and C, filled arrowhead), with lengths of approximately 20–30 μm and 20–50 μm in the females and males, respectively, are observed. Moreover, the sensilla have multiparous structures on the surface and lack a socket at the base, suggesting that they are olfactory sensilla. Female-specific contact chemosensory structures were not observed in the middle or distal portions of the antenna. Although we do not have information about molecular receptors and receptor neurons for antennal chemosensory organs exhibiting oviposition behavior, our SEM observations suggest that females may detect contact chemicals using sensilla chaetica and volatiles by sensilla basiconica and sensilla trichodea.

### Identification of oviposition stimulants

**Figure 2:**
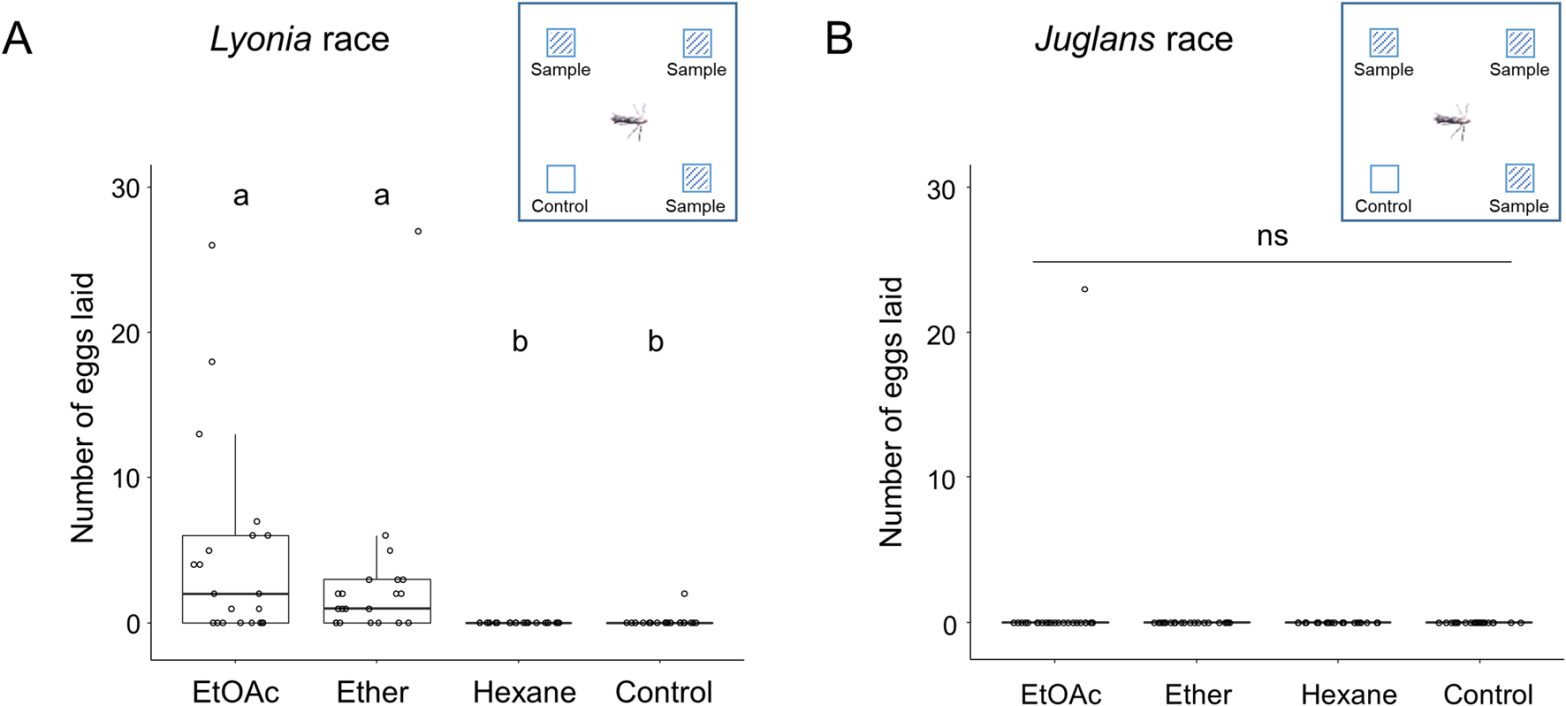
Oviposition responses of *Lyonia* and *Juglans* race females to leaf extracts of *Lyonia ovalifolia*. (**A**) Number of eggs laid by *Lyonia* race females on cover glasses treated with or without leaf extract using different solvents in four-choice assays. Different letters indicate statistically significant differences among the treatments by Steel–Dwass test, *p < 0.01 (*n* = 21). (**B**) Oviposition responses of *Juglans* race females on cover glasses treated with or without leaf extract using different solvents in four-choice assays. No significant difference was observed among the treatments by Steel–Dwass test (*n* = 24).

First, we examined whether gravid females of the *Lyonia* and *Juglans* races laid eggs in response to the *L*. *ovalifolia* leaf extract. We designed a bioassay in which an individual mated female was allowed to select cover glasses treated either with sample(s) or solvent (EtOH) for oviposition in a plastic container. After overnight, the number of eggs on and around the cover glasses was counted to evaluate the oviposition stimulant activity. The *Lyonia* race females laid eggs on the cover glasses treated with the ethyl acetate (EtOAc) or ether extract, but not with the hexane extract. (Fig. 2A, Table S2). The numbers of eggs laid on the cover glasses varied greatly among individual females (Table S2). In contrast, none of the *Juglans* race females, except one, laid eggs on a cover glass treated with L. *ovalifolia* leaf extracts; the sole exception responded to the EtOAc extract (*n* = 24) (Fig. 2B, Table S3). The EtOAc extract was further fractionated using silica gel column chromatography to identify the oviposition stimulants of the *Lyonia* race (Fig. 3A). Half of the tested females (16 of 32) laid eggs on the cover glasses treated with the separated fractions or untreated. Comparing the percentage of eggs laid by individual females on each cover glass, the fraction eluted with acetone (Fr. AC) exhibited significant activity (Fig. 3B, Table S4). Thus, the acetone fraction was further fractionated using ODS column chromatography. Of the tested females, 30% (6 of 20) laid eggs on the cover glasses treated with the separated fractions or untreated. Among the ODS eluates, a mixture of fractions (Fr. 4–6) exhibited comparatively higher activity than another mixture of Fr. 1–3; however no significant activity was observed between Fr. 4–6 and the controls. (Fig. 3C, Table S5). It is known that lepidopteran species generally utilize highly polar compounds^35–37^, including specific sugar and amino acid derivatives as oviposition stimulants, therefore, a mixture of a highly polar fraction (Fr. A) prepared from the EtOH extract of *Lyonia* leaves (Fig. 3D) and each sample was tested. During the two-choice assays, almost all females did not lay eggs on the cover glasses. The remainder laid eggs on either a cover glass treated with a sample or that was untreated. (Tables S6 and S7). Comparing the percentage of oviposited females, the mixture (Fr. 4–6 + Fr. A) exhibited significant activity (Fig. 3E, Table S6). Among the ODS eluates, Fr. 6 mixed with Fr. A (Fr. 6 + Fr. A) exhibited oviposition stimulant activity. From Fr. 6, three compounds, **1**, **2**, and **3**, were isolated as the main components. The quantities of **1**, **2**, and **3** were 0.40, 0.29, and 0.21 mg per gram leaf. None of the compounds exhibited activity on its own, but compounds **1** and **2** exhibited significant stimulant activity when mixed with Fr. A (Fig. 3F, Table S7). We further examined whether the mixture of **1** and **2** increased the oviposition stimulant activity but did not observe a marked enhancement of the oviposition response upon mixing the stimulants (Fig. 3F, Table S7). We also observed that none of the *Juglans* race responded to the mixture of **1** and Fr. A (*n* = 21) (Table S8).

**Figure ﻿3:**
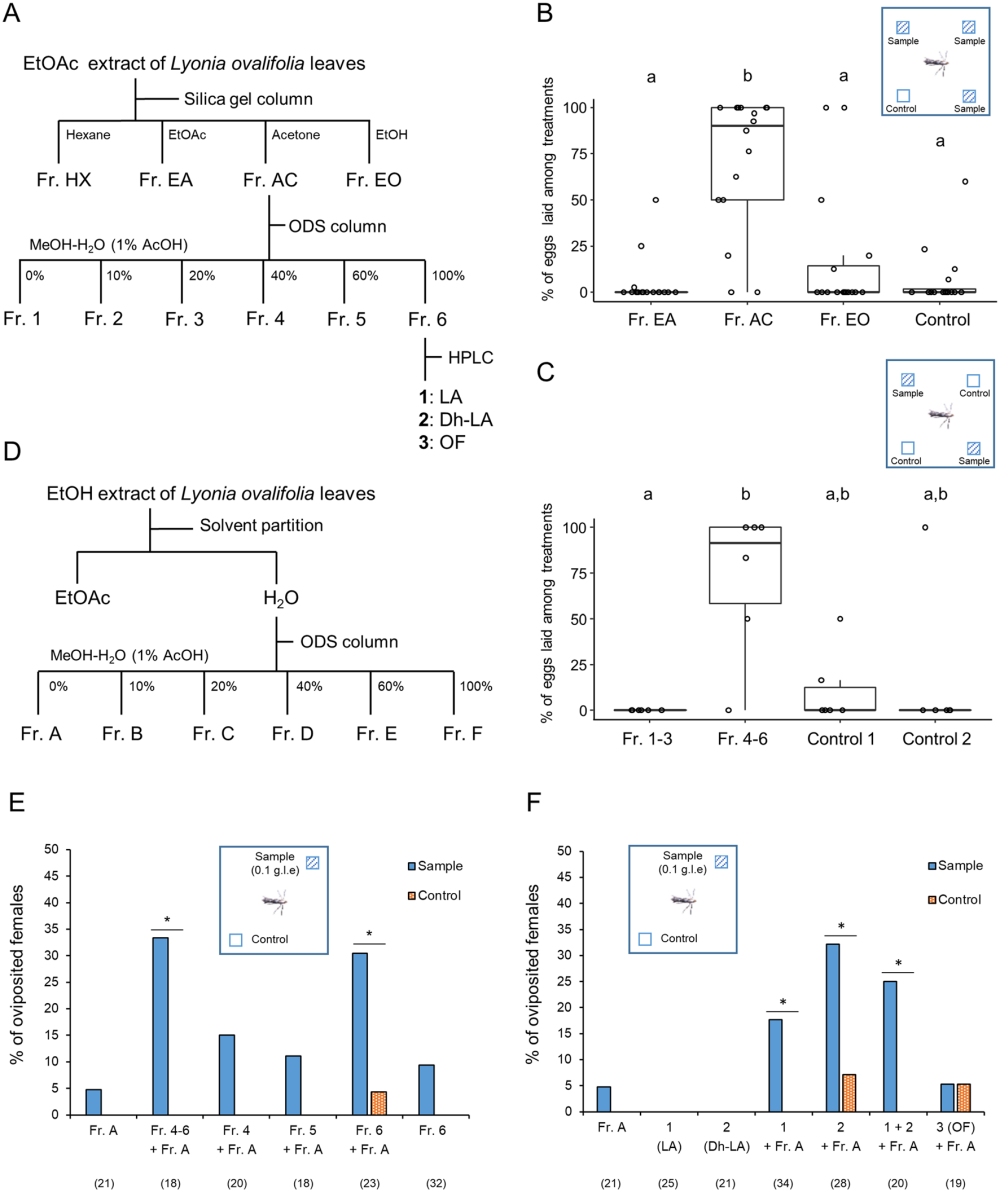
Separation procedure and oviposition stimulant activities of *Lyonia ovalifolia* fractions for *Lyonia* race females. (**A**) Separation procedure to isolate triterpene glycosides. (**B**) Percentage of the eggs laid by individual females on cover glasses treated with or without fractions separated using silica gel column chromatography. Different letters indicate statistically significant differences among the treatments by Steel–Dwass test, *p < 0.01 (*n* = 16). (**C**) Percentage of the eggs laid by individual females on cover glasses treated with or without fractions separated using ODS column chromatography. Different letters indicate statistically significant differences among the treatments by Steel–Dwass test, *p < 0.01 (*n* = 6). (**D**) Separation procedure to prepare the highly polar fraction (Fr. A). (**E**) Percentage of *Lyonia* race females oviposited on cover glasses treated with or without samples in two-choice assays. A significant difference between the sample and control was analyzed within each two-choice set by Fisher’s exact test, *p < 0.05. Numbers in parentheses represent the number of tested animals. (**F**) Percentage of *Lyonia* race females oviposited on cover glasses treated with or without samples in two-choice assays. A significant difference between the sample and control was analyzed within each two-choice set by Fisher’s exact test, *p < 0.05. Numbers in parentheses represent the number of tested animals.

**Figure 4:**
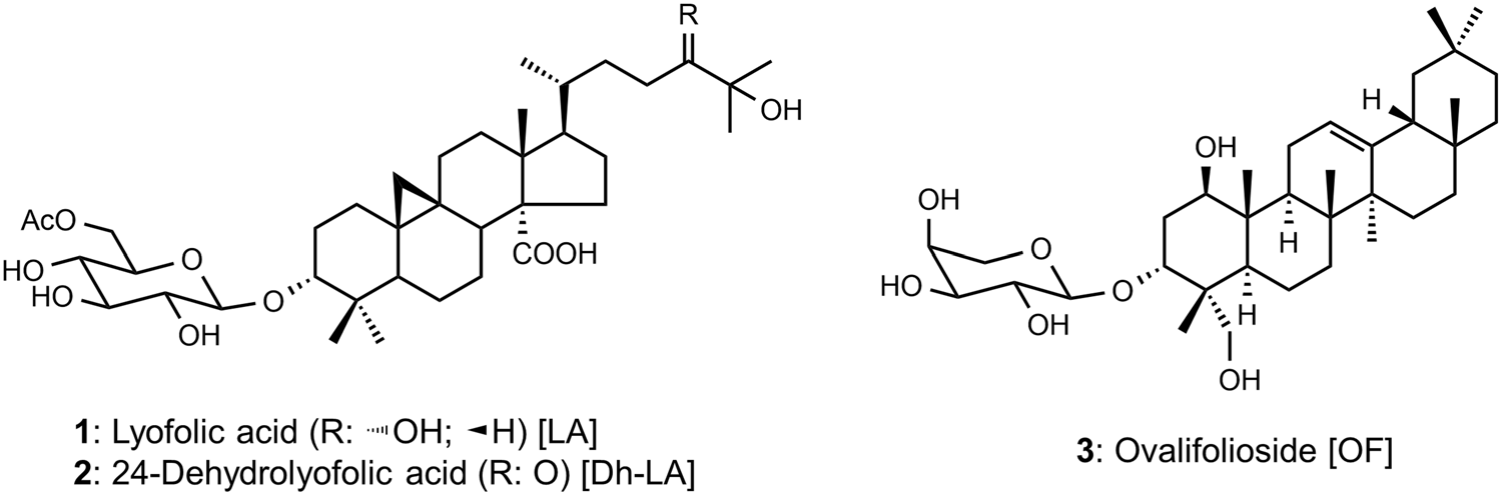
Structures of triterpenoid glycosides (**1**–**3**) isolated from leaves of *Lyonia ovalifolia*.

Compounds **1** and **3** were identified as lyofolic acid (LA) and ovalifolioside (OF), respectively (Fig. 4), by comparison with the spectral data previously reported of compounds isolated from the leaves of *L*. *ovalifolia*^38–42^ (Table S9; supplementary materials). The molecular formula of **2** was determined to be C_38_H_60_O_11_ by HR-ESI–MS analysis. The ^1^H NMR spectrum revealed two doublet signals of methine protons (δ 0.60 and 0.38) at a cyclopropane ring, eight signals of protons (δ 4.96–3.73), adjacent oxygens including an anomeric proton (δ 4.82), and seven signals of methyl protons (δ 2.00, 1.55, 1.55, 1.20, 1.20, 1.00, and 0.95), suggesting that **2** is a cycloartane-type triterpene glycoside. The ^13^C NMR revealed 38 signals, including overlapped signals (δ 27.3) derived from the methine and methyl carbons. The aforementioned ^1^H and ^13^C NMR spectra were remarkably similar to those of compound **1**. The notable differences were the signals at δ 3.02 and 2.95 of **2** in the ^1^H NMR spectrum, and the signal at δ 216.2 of **2** instead of that at δ 79.1 of **1** in the ^13^C NMR spectrum, suggesting the presence of a carbonyl group at the 24-position of **2** instead of the hydroxyl group at the same position of **1**. Furthermore, the assignment of proton and carbon signals by ^1^H-^1^H COSY, HSQC, and HMBC analyses supported the structure of **2** as 24-dehydrolyofolic acid. To confirm the structure of **2**, the hydroxyl group at the 24-position in **1** was oxidized to a 24-dehydro derivative using platinum IV oxide, as described in a previous paper^43^. The ^1^H-NMR spectrum of **2** was identical to that of the oxidized product of **1** (Fig. S1), therefore, **2** was determined to be 24-dehydrolyofolic acid (Dh-LA) (Fig. 4).

### Oviposition on non-host leaves by treatment with the stimulants

**Figure 5:**
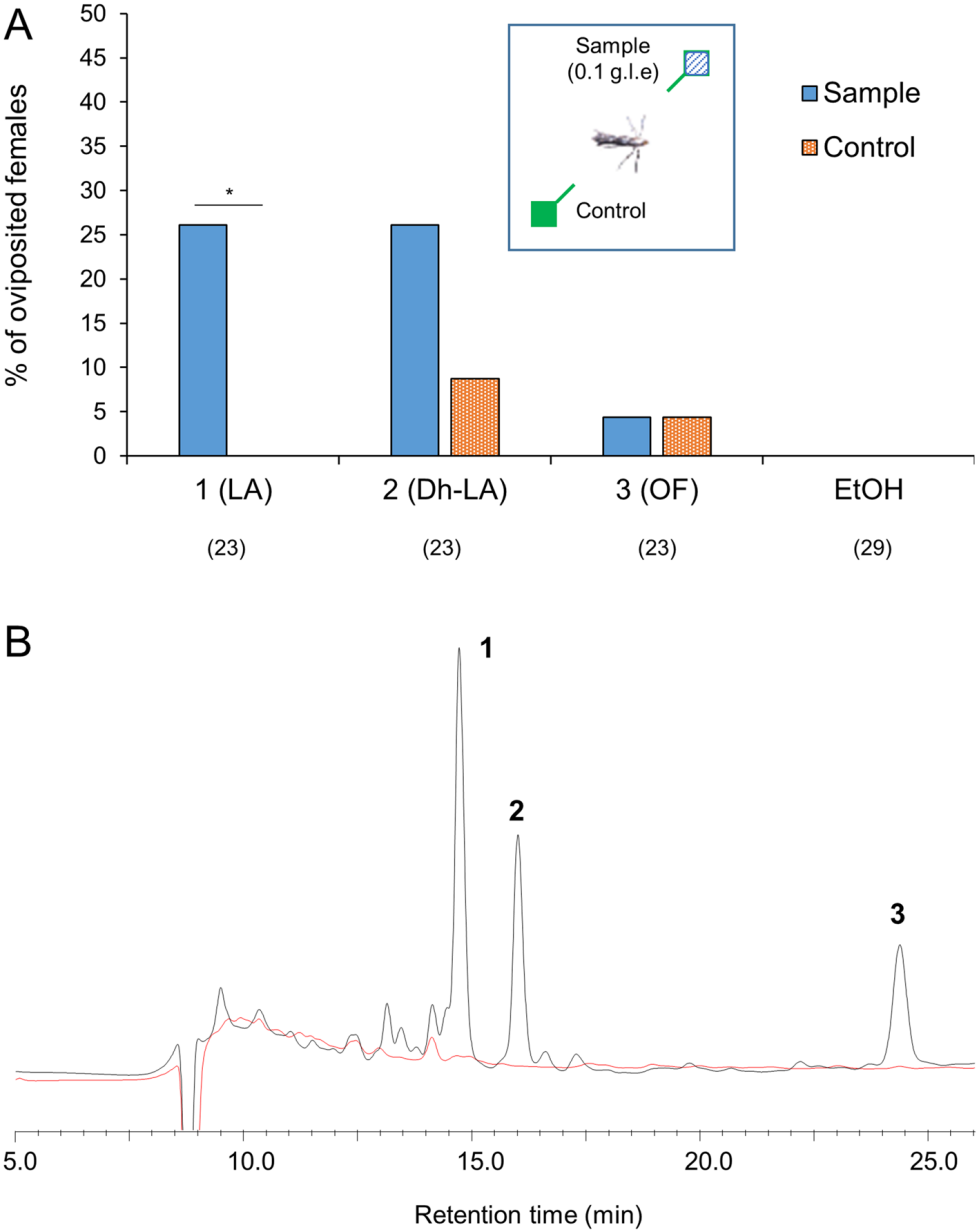
Oviposition of *Lyonia* race females on *Juglans* leaves treated with the *Lyonia*-specific triterpenoids. (**A**) Percentage of *Lyonia* race females oviposited on pieces of leaves of *Juglans regia* treated with or without samples in two-choice assays. A significant difference between the sample and control was analyzed within each two-choice set by Fisher’s exact test *p < 0.05. Numbers in parentheses represent the number of tested animals. (**B**) HPLC analyses of Fr. 6 prepared using ODS column chromatography. Black and red lines indicate fractions prepared from extract of *L*. *ovalifolia* and *Juglans mandshurica*, respectively. Each 1 mg of fraction (0.49- and 0.39-g leaf equivalents for *L*. *ovalifolia* and *J*. *mandshurica*, respectively) was analyzed using HPLC.

Because the *Lyonia* race females never oviposit on *Juglans* leaves, we assumed that *Juglans* leaves do not contain essential oviposition stimulant(s) for *Lyonia* race females. It is also possible that *Juglans* leaves contain deterrent compounds against *Lyonia* race female oviposition. To evaluate these hypotheses, *Lyonia* race females were exposed to the fresh leaves of *Juglans regia* treated with the triterpenoid glycosides, either LA (**1**), Dh-LA (**2**), or OF (**3**), and untreated leaves (solvent only) (Fig. 5A, Table S10). Oviposition was elicited by treatment with LA (**1**) but not by the untreated control. We also observed that treatment with Dh-LA (**2**) elicited a higher oviposition response than the untreated control; however, no statistically significant difference was observed between the responses. The treatment with OF (**3**) elicited a relatively low oviposition response at the same level as the untreated control. To determine whether the *Juglans* leaves contain *Lyonia*-specific glycosides, we compared the chemical profiles of fractions prepared from *Lyonia* and *Juglans*-extracts. Fr. 6 was prepared from the EtOAc extract of *Juglans* leaves according to the separation procedure of the *Lyonia*-extract. In contrast to *Lyonia*-Fr. 6, the corresponding *Juglans* fraction did not contain noteworthy compounds, including LA (**1**), Dh-LA (**2**), and OF (**3**) (Fig. 5B).

## Discussion

In the present study, we characterized the *Lyonia*-specific triterpenoid glycosides as oviposition stimulants for the derived host race, the *Lyonia* race. Behavioral observation and ablation experiments indicate that the antennae are crucial for the detection of oviposition stimulants. The ablation of one of a pair of antennae did not affect oviposition, suggesting that physical damage does not reduce oviposition. A small number of females laid eggs on leaves, even when both antennae and foreleg tarsi were ablated, indicating that another chemosensory organ(s) and/or other senses are likely involved in the induction of oviposition. Considering the water-soluble, but not volatile, properties of these triterpenoid glycosides, the identified oviposition stimulants must be recognized via contact chemoreception. To find the contact chemosensory organs of females, we performed SEM observations of the middle and distal portions of the antennae because these portions are especially important for tapping the oviposition sites. It was revealed that both female and male antennae had sensilla chaetica, generally considered contact chemosensory hair in various insect species including moths^34^. No female-specific contact chemosensory structure was observed on these portions of the antennae. Therefore, we speculate that the receptor neurons housed in the sensilla chaetica may be tuned to detect oviposition stimulants in females. In lepidopteran species, responses of antennal contact chemosensilla to oviposition stimulants have been observed by electrophysiological recordings in the monarch butterfly, *D*. *plexippus*^33^. Likewise, the analysis of electrophysiological responses is necessary to confirm that the gustatory sensilla on the surface of the antennae can detect oviposition stimulants in *A*. *transecta*.

Previous studies have shown that the similarity of the chemical profiles between plants is a factor that is critical for determining host shifts by phytophagous insects^11,12,24^. For example, identical or similar secondary metabolites, such as hydroxycinnamic acids, flavonoids, and glucosinolates, contained in different host plant taxa are commonly utilized as oviposition stimulants in the related lepidopteran species^24,44,45^. Thus, secondary metabolites within the common classes appear to be used by the related species as host recognition cues during host shifts and the expansion associated with speciation. However, these studies have dealt with related species that had already completed speciation in the past; therefore, it is impossible to identify chemical factors that directly play critical roles during host shifting. In contrast, this study focused on host race formation, probably where speciation is in progress. We explored the chemosensory cues underlying the different oviposition preferences between the host races. We identified oviposition stimulants of the *Lyonia* race as specific triterpenoid glycosides contained exclusively in the derivative plant, *L*. *ovalifolia*. The *Juglans* race did not respond to the oviposition stimulants LA. Therefore, ancestors of the *Lyonia* race probably acquired the ability to detect newly encountered triterpenoid glycosides as oviposition stimulants.

Notably, the dual use of ancestral and derived host plants has not been observed in *A*. *transecta*^26^. This suggests that the acquisition of responsiveness to *Lyonia*-specific oviposition stimulants and the loss of the ability to recognize *Juglans* leaves as host plants occurred simultaneously, thereby promoting the formation of the *Lyonia* race. In this process, the novel stimulants might have become necessary for recognizing the current host, thereby favoring the loss of recognition of *Juglans* leaves. Otherwise, the response spectra of a pre-existing receptor for eliciting oviposition behavior might have shifted from chemicals in *Juglans* leaves to LA, or a receptor responding to oviposition deterrents in *Lyonia* leaves may have been lost.

The mechanisms underlying specific alterations in the chemosensory systems in host plant preference have been elucidated by studies on the stringent specialist *D*. *sechellia*, which exclusively utilizes the toxic morinda fruit as its host plant, in comparison with its sibling generalist species, *D*. *melanogaster* and *D*. *simulans*^46^. The morinda fruit contains toxic short-chain fatty acids that induce the oviposition of *D*. *sechellia* females. Multiple alterations in the chemosensory system to detect the chemicals in the morinda fruit have been reported in *D*. *sechellia*. The antennal sensilla of the *Drosophila* species are divided into subgroups. A subgroup of the antennal sensilla has been replaced by sensilla that respond to the characteristic volatiles of the morinda fruit in *D*. *sechellia*, which could cause the behavioral shift preferentially to specific volatiles via increased antennal sensitivity^47,48^. Morphological changes and increased neuropil modules in the brain responsible for receiving input from olfactory neurons that tune to the specific volatiles of the morinda fruit have also been observed in *D*. *sechellia*^47^. At the molecular levels, loss-of-function mutations in multiple gustatory receptors presumed to detect bitter compounds have been found in *D*. *sechellia*^49^. The importance of the expression of odorant-binding protein (OBP) responsible for the host preference has also been demonstrated^50^. Two OBPs expressed in the gustatory neurons of the leg tarsi are involved in the specific detection of toxic acids. While the two OBPs function to avoid the toxic acids in the generalists, *D*. *melanogaster* and *D*. *simulans*, a mutation in the upstream regulatory region of one of the OBPs results in the loss of OBP function, which could cause oviposition preference by overriding the gustatory avoidance in *D*. *sechellia*. Therefore, the loss of detection of oviposition deterrents due to a mutation of chemosensory genes may cause the host shift to the derivative plant in the *Lyonia* race.

Neither the specific triterpenoid glycosides, LA and Dh-LA, nor the highly polar fraction Fr. A exhibited oviposition activity on its own. However, the mixture of Fr. A with **1** or **2** elicited oviposition in the *Lyonia* race. Therefore, a synergistic effect on oviposition was observed in the *Lyonia* race. A similar combination system with multiple components has been well characterized in papilionid butterflies, and such a system enables butterflies to strictly distinguish limited host plant species^36,37,51^. As high polar components, several classes of compounds derived from primary metabolites such as cyclitols and amino acid derivatives have been characterized as oviposition stimulants via tarsal contact chemoreceptors^35–37^. Considering the two results of the experiments for *Lyonia* race females: (i) the combination of LA and Fr. A, but not LA alone, elicited oviposition; (ii) LA treatment alone on *Juglans* leaves elicited oviposition, *Juglans* leaves probably contained an oviposition stimulant. Thus, it would be interesting if the oviposition stimulant is commonly present in *Juglans* and *Lyonia* leaves. The second result regarding the elicited oviposition on *Juglans* leaves further offers two possible explanations: (i) *Lyonia* race females do not lay eggs on *Juglans* leaves owing to the lack of specific oviposition stimulant(s), and not because of the presence of oviposition deterrents, or (ii) the activity of oviposition stimulants overcomes that of oviposition deterrents contained in *Juglans* leaves. The identification of the presumed common oviposition stimulant will provide further insights into the common and differential detection of plant chemicals in the two host races.

When a host shift occurs, parental oviposition preference and larval performance must coincide with each other^52,53^. Notably, one exception was observed, in which a *Juglans* race female laid eggs on a cover glass treated with *Lyonia* leaf extract. The occasional erroneous response to non-host chemicals and subsequent oviposition mistakes may be an initial step in host shifting. Oviposition mistakes have been reported in various phytophagous insects, which have been considered a potential factor for a shift to a new plant species^54,55^. If a *Juglans* race female pre-equipped with the ability to overcome defensive substances through novel mutations occasionally laid eggs on *Lyonia* leaves, its progeny could survive on the non-host leaves. Subsequently, females preferring to oviposit on *Lyonia* probably appeared in the population where larvae were able to survive on *Lyonia* leaves^26^. Following this hypothesis, genetic analysis using hybridization of the two host races of *A*. *transecta* has shown that oviposition preference and larval performance fall under different genetic controls^27^. A combination of a chemical approach and genetic analysis could lead to the identification of the genes required for the changes in the adult recognition of a new host plant and for the capacity of the larvae to metabolize toxic compounds contained within the plant; thus, furthering our understanding of the mechanisms underlying a host shift at the molecular level.

## Materials and methods

### Insects

Larvae of *A*. *transecta* in mined leaves were collected from the host plants, *J*. *mandshurica*, and *L*. *ovalifolia* in the suburbs of Kyoto, Japan. The larvae were maintained at approximately 25 °C under a photoperiod regimen of 16-h light/8-h dark, as described previously^56^. Each eclosed female was crossed with a male in a 50-mL plastic tube containing a host leaf. After the confirmation of oviposition on their host leaves, mated gravid females were used for the bioassay. All methods were performed in accordance with the relevant guidelines and regulations.

### Scanning electron microscopy (SEM)

Heads of female and male were kept in 40% ethanol, and the scales of middle and distal portions of antennal segments were removed. The heads were processed ethanol series (50, 60, 70, 80, 85, 90, 95, 99, and 100%) before being critical point dried in a Tousimis Samdri-795 (Rockville, MD, USA). The antennae were removed from the head and mounted on an SEM stub with carbon tape. The specimens were coated with AuPd (either Hammer 6.2 Sputtering System, Anatech Ltd., Springfield, VA, USA, or Cressington 108 sputter coater, Watford, UK) before imaging in JEOL JSM-5900LV (Tokyo, Japan), Hitachi SU3900 SEM (Tokyo, Japan) or a FEI Verios 460L (Hillsboro, Oregon, USA). In this study, we classified the observed sensory hairs according to morphological characteristics but did not analyze the details of the distribution and function of the sensilla.

### Extraction of *L*. *ovalifolia* leaves

Fresh leaves of *L*. *ovalifolia* (50 g) collected from the suburbs of Kyoto were thoroughly extracted with hexane, ether, or else EtOAc for 1 week. Each extract was concentrated in vacuo and dissolved in 20 mL ethanol. The extract (20 μL) was used for the oviposition stimulant bioassay, as described later.

### Isolation of oviposition stimulants

Fresh leaves of *L*. *ovalifolia* (500 g) were extracted with EtOAc as described above. The EtOAc extract (5.37 g: 400 g leaf equivalent (g.l.e.)) was chromatographed on a silica gel column (150 g of Wakogel C-200, Wako Pure Chemical Co., Osaka, Japan), eluted successively with hexane (Fr. HX), EtOAc (Fr. EA), acetone (Fr. AC), and ethanol (Fr. EO) (yield: Fr. HX: 16.7 mg; Fr. EA: 2.53 g; Fr. AC: 2.10 g; Fr. EO: 86.7 mg). Fr. AC (1.58 g: 300 g.l.e.) was subsequently chromatographed on a reverse-phase ODS column (30 g of Cosmosil 140C18-OPN, 140 μm, Nacalai Tesque, Kyoto, Japan) and eluted successively with H_2_O (Fr. 1), 10% methanol (Fr. 2), 20% methanol (Fr. 3), 40% methanol (Fr. 4), 60% methanol (Fr. 5), and 100% methanol (Fr. 6) (yield: Fr. 1: 86.2 mg; Fr. 2: 42.5 mg; Fr. 3: 85.9 mg; Fr. 4: 653 mg; Fr. 5: 184 mg; Fr. 6: 613 mg). Fr. 6 was chromatographed on a reverse-phase HPLC column (YMC-Pack Pro C18, AS-343, 250 × 10 mm i.d., YMC Ltd., Kyoto, Japan) eluted with 95% methanol in 1% acetic acid (aq.) at a flow rate of 1.5 mL/min. Compounds **1**, **2**, and **3** were isolated at retention times of 14.7, 16.0, and 24.4 min, respectively, monitoring with a refractive index detector (RID-10A, Shimadzu, Kyoto, Japan).

### Preparation of a highly polar fraction (Fr. A)

Fresh leaves of *L*. *ovalifolia* (110 g) were thoroughly extracted with ethanol (EtOH) for 1 year. The EtOH extract (3.03 g: 110 g.l.e.) was dissolved in water (H_2_O) and partitioned with EtOAc. The H_2_O layer (1.52 g: 50 g.l.e.) was chromatographed on a reverse-phase ODS column (20 g of Cosmosil 140C18-OPN, 140 μm, Nacalai Tesque), and elution with H_2_O yielded a highly polar fraction (Fr. A) (809 mg).

### Preparation of the Fr. 6 from *Juglans* leaves

Fresh leaves of *J*. *mandshurica* (100 g) that were also collected from a suburb of Kyoto were extracted with EtOAc for 2 years. The EtOAc extract was separated in a manner similar to the separation of *Lyonia* extract, as described above. The corresponding Fr. 6 (255 mg) was obtained from 100 g of leaves.

### Ablation experiment

Gravid females were anaesthetized using triethylamine, and then the antennae or tarsi of the forelegs were dissected at their bases using a razor blade. Each treated female was allowed to oviposit on a *Lyonia* leaf in a plastic container (105 × 105 × 50 mm) overnight. The percentages of females laid eggs on a *Lyonia* leaf were calculated.

### Oviposition stimulant bioassay

An extract of *Lyonia* leaves or EtOH (control) was applied to cover glasses (18 × 18 mm Trophy, Matsunami Glass Inc., Ltd., Osaka, Japan), and the solvent was dried at room temperature. Each gravid female was allowed to oviposit on four cover glasses, including the control. The cover glasses were placed in a plastic container (105 × 105 × 50 mm) overnight. We counted the number of eggs on the cover glasses and in the surrounding area within 5 mm of the cover glasses (i.e., 23 × 23 mm). First, the numbers of eggs laid by the individual *Lyonia* and *Juglans* race females in the presence of *Lyonia ovalifolia* leaf extracts were analyzed to compare responses to the extracts between the host races. Gravid *Lyonia* race females that responded to the EtOAc extract were used in the following experiments. After fractionation of the *Lyonia*-EtOAc or -EtOH extracts by column chromatography, each sample solution or EtOH (control) was applied to cover glasses, and then each gravid female was allowed to oviposit on either four or two cover glasses, as described above. In four-choice assays, the percentages of eggs on each cover glass to the total number of eggs laid on the four cover glasses were calculated to normalize the variation in egg number laid by individual females. In two-choice assays, the percentages of oviposited females for each cover glass were calculated. Additionally, *Juglans* leaves (*J*. *regia*) were cut into 18 × 18 mm squares with a leafstalk (ca. 10 mm) (Fig. 5A) and treated with a test compound or solvent. Each gravid female was allowed to oviposit on the differently treated leaves, as described above. The percentages of oviposited females for each *Juglans* leaf were calculated. Each cover glass or *Juglans*-leaf was treated with an extract, fraction, or compound at a concentration of 0.1 g.l.e.

### Oxidation of compound 1 into 2

Compound **1** (5 mg) dissolved in a mixture of H_2_O and methanol (1:1) was oxidized by bubbling oxygen gas with a catalytic quantity of reduced platinum (IV) oxide (ca. 50 mg) (Wako Pure Chemical Co.)^43^. The oxidized product was purified via HPLC under the aforementioned condition to isolate **2**.

### Instruments used for chemical analysis

The HR-ESI–MS spectra were measured using an Exactive Plus mass spectrometer (Thermo Fisher Scientific, Bremen, Germany). The ^1^H-NMR and ^13^C-NMR spectra were measured using a Bruker Avance III 500 spectrometer (Bruker Daltonics, Bremen, Germany) with TMS as an internal standard. Optical rotations were measured using a JASCO P-1010 spectropolarimeter (Jasco Co., Tokyo, Japan).

### Spectral data

**1** (lyofolic acid: 3α-(6ʹ-*O*-acetyl-d-β-glucopyranosyloxy)-24(*R*),25-dihydroxy-9,19-cyclolanostan-30-oic acid): [α]_D_^25^**–**18.8 (*c* = 1.0, CH_3_OH). ^1^H-NMR (C_5_D_5_N): δ 4.96 (1H, dd, *J* = 11.6, 2.0 Hz, H-6ʹa), 4.82 (1H, d, *J* = 9.2 Hz, H-1ʹ), 4.81 (1H, dd, *J* = 11.6, 6.1 Hz, H-6ʹb), 4.24 (1H, dd, *J* = 8.8, 8.7 Hz, H-3ʹ), 4.07 (1H, dd, *J* = 9.6, 8.7 Hz, H-4ʹ), 4.00 (1H, ddd, *J* = 9.6, 6.1, 2.0 Hz, H-5ʹ), 3.97 (1H, dd, *J* = 9.2, 8.8 Hz, H-2ʹ), 3.79 (1H, d, *J* = 9.2 Hz, H-24), 3.73 (1H, brs, H-3), 2.66 (1H, m, H-11a), 2.55 (1H, m, H-15a), 2.51 (1H, m, H-2a), 2.50 (1H, m, H-16a), 2.26 (1H, dd, *J* = 12.7, 3.7 Hz, H-5), 2.16 (1H, m, H-12a), 2.12 (1H, m, H-1a), 2.05 (1H, m, H-17), 2.00 (3H, s, CH_3_CO–), 1.91 (1H, m, H-12b), 1.88 (1H, m, H-22a), 1.85 (2H, m, H-23), 1.85 (1H, m, H-1b), 1.78 (1H, dd, *J* = 11.9, 5.5 Hz, H-8), 1.70 (1H, m, H-22b), 1.69 (1H, m, H-20), 1.57 (1H, m, H-16b), 1.55 (1H, m, H-7), 1.54 (3H, s, H-26/27), 1.51 (3H, s, H-26/27), 1.50 (1H, m, H-6a), 1.38 (1H, m, H-15b), 1.33 (1H, m, H-11b), 1.24 (3H, s, H-18), 1.20 (3H, s, H-29), 1.16 (1H, m, H-2b), 1.05 (3H, d, *J* = 5.8 Hz), 1.01 (3H, s, H-28), 0.82 (1H, m, H-6b), 0.61 (1H, d, *J* = 3.5 Hz, H-19a), 0.39 (1H, d, *J* = 3.5 Hz, H-19b). ^13^C-NMR (C_5_D_5_N): see Table S9. HR-ESI–MS *m*/*z* [M + Na]^+^: calcd. for C_38_H_62_O_11_Na 717.4190; found, 717.4185.

**2** (24-dehydrolyofolic acid: 3α-(6ʹ-*O*-acetyl-d-β-glucopyranosyloxy)-24-one-25-hydroxy-9,19-cyclolanostan-30-oic acid): [α]_D_^25^**–**23.6 (*c* = 1.0, CH_3_OH). ^1^H-NMR (C_5_D_5_N): δ 4.96 (1H, dd, *J* = 11.6, 2.0 Hz, H-6ʹa), 4.82 (1H, d, *J* = 7.9 Hz, H-1ʹ), 4.81 (1H, dd, *J* = 11.6, 6.2 Hz, H-6ʹb), 4.24 (1H, dd, *J* = 8.8, 8.7 Hz, H-3ʹ), 4.06 (1H, dd, *J* = 9.6, 8.7 Hz, H-4ʹ), 4.00 (1H, ddd, *J* = 9.6, 6.2, 2.0 Hz, H-5ʹ), 3.96 (1H, dd, *J* = 8.8, 7.9 Hz, H-2ʹ), 3.73 (1H, brs, H-3), 3.02 (1H, m, H-23a), 2.95 (1H, m, H-23b), 2.65 (1H, m, H-11a), 2.54 (1H, m, H-15a), 2.52 (1H, m, H-2a), 2.44 (1H, m, H-16a), 2.25 (1H, dd, *J* = 12.7, 3.8 Hz, H-5), 2.12 (1H, m, H-12a), 2.11 (1H, m, H-1a), 2.06 (1H, m, H-22a), 2.01 (1H, m, H-17), 2.00 (3H, s, CH_3_CO-), 1.88 (1H, m, H-12b), 1.85 (1H, m, H-1b), 1.77 (1H, dd, *J* = 11.9, 5.4 Hz, H-8), 1.60 (1H, m, H-20), 1.58 (1H, m, H-7), 1.57 (1H, m, H-16b), 1.55 (3H, s, H-26/27), 1.55 (3H, s, H-26/27), 1.49 (1H, m, H-6a), 1.48 (1H, m, H-22b), 1.37 (1H, m, H-15b), 1.31 (1H, m, H-11b), 1.20 (3H, s, H-18), 1.20 (3H, s, H-29), 1.16 (1H, m, H-2b), 1.00 (3H, s, H-28), 0.95 (3H, d, *J* = 6.5 Hz, H-21), 0.82 (1H, m, H-6b), 0.60 (1H, d, *J* = 3.6 Hz, H-19a), 0.38 (1H, d, *J* = 3.6 Hz, H-19b). ^13^C-NMR (C_5_D_5_N): see Table S9. HR-ESI–MS *m*/*z* [M + Na]^+^: calcd. for C_38_H_60_O_11_Na 715.4033; found, 715.4031.

**3** (ovalifolioside: 3β-*O*-α-l-arabinopyranosyloxy-olean-12-ene-1β,23-diol): [α]_D_^24^ + 54.0 (*c* = 1.0, CH_3_OH). ^1^H-NMR (C_5_D_5_N): δ 7.14 (1H, brs, –OH), 6.59 (1H, brs, –OH), 6.28 (1H, brs, –OH), 6.11 (1H, brs, –OH), 5.67 (1H, d, *J* = 5.4 Hz, –CH_2_OH), 5.38 (1H, t-like, H-12), 5.00 (1H, d, *J* = 7.2 Hz, H-1ʹ), 4.45 (1H, m, H-1), 4.44 (1H, dd, *J* = 8.9, 7.2 Hz, H-2ʹ), 4.38 (1H, d, *J* = 10.6 Hz, H-23a), 4.25 (1H, ddd, *J* = 3.2, 2.5, 2.1 Hz, H-4ʹ), 4.24 (1H, dd, *J* = 12.5, 2.5 Hz, H-5aʹ), 4.06 (1H, dd, *J* = 8.9, 3.2 Hz, H-3ʹ), 3.87 (1H, m, H-3), 3.77 (1H, d, *J* = 10.6 Hz, H-23b), 3.69 (1H, dd, *J* = 12.5, 2.1 Hz, H-5bʹ), 3.19 (1H, m, H-11a), 2.84 (1H, m, H-2a), 2.49 (1H, m, H-11b), 2.47 (1H, m, H-2b), 2.17 (1H, dd, *J* = 11.2, 6.2 Hz, H-9), 2.04 (1H, dd, *J* = 12.4, 3.5 Hz, H-18), 1.99 (1H, dd, *J* = 13.4, 4.2 Hz, H-16a), 1.84 (1H, m, H-6a), 1.79 (1H, m, H-15a), 1.75 (1H, m, H-19a), 1.73 (1H, m, H-5), 1.71 (1H, m, H-7a), 1.61 (1H, m, H-6b), 1.46 (1H, m, H-22a), 1.38 (3H, s, H-25), 1.37 (1H, m, H-21a), 1.37 (1H, m, H-7b), 1.26 (3H, s, H-27), 1.23 (1H, m, H-22b), 1.15 (3H, s, H-26), 1.10 (1H, m, H-19b), 1.10 (1H, m, H-21b), 1.02 (3H, s, H-24), 0.96 (1H, m, H-15b), 0.94 (3H, s, H-28), 0.90 (3H, s, H-29), 0.88 (3H, s, H-30), 0.79 (1H, m, H-16b).

^13^C-NMR (C_5_D_5_N): see Table S9. HR-ESI–MS *m*/*z* [M + Na]^+^: calcd. for C_35_H_58_O_7_Na 613.4080; found, 613.4077.

## Statistical analysis

Statistical analyses were performed using the R software version 3.6.1 (https://www.r-project.org/). Pairwise comparisons using the Fisher’s exact test with Holm corrections for multiple testing were performed using the “fisher.multcomp” function in the R package “RVAideMemoire”.

## Supplementary Information

Supplementary Video 1.

Supplementary Information 1.

## Data Availability

All data are included within the paper and its Supporting Information files.

## References

[1] Schoonhoven L, van Loon J, Dicke M (2005). Insect–plant biology.

[2] Bernays, E. A. & Chapman, R. F. Behavior: The process of host-plant selection. in *Host-Plant Selection by Phytophagous Insects* 95–165 (Chapman & Hall, 1994).

[3] Orsucci M (2016). Host specialization involving attraction, avoidance and performance, in two phytophagous moth species. J. Evol. Biol..

[4] Jaenike J (1990). Host specialization in phytophagous insects. Annu. Rev. Ecol. Syst..

[5] Fordyce JA (2010). Host shifts and evolutionary radiations of butterflies. Proc. R. Soc. B Biol. Sci..

[6] Endara MJ (2017). Coevolutionary arms race versus host defense chase in a tropical herbivore–plant system. Proc. Natl. Acad. Sci. U.S.A..

[7] Ehrlich PR, Raven PH (1964). Butterflies and plants: A study in coevolution. Evolution (NY).

[8] Becerra JX, Venable DL (1999). Macroevolution of insect-plant associations: The relevance of host biogeography to host affiliation. Proc. Natl. Acad. Sci. U.S.A..

[9] Drès M, Mallet J (2002). Host races in plant-feeding insects and their importance in sympatric speciation. Philos. Trans. R. Soc. B Biol. Sci..

[10] Berlocher SH, Feder JL (2002). Sympatric speciation in phytophagous insects: Moving beyond controversy?. Annu. Rev. Entomol..

[11] Janz N, Nylin S (1998). Butterflies and plants: A phylogenetic study. Evolution (NY).

[12] Muto-Fujita A (2017). Data integration aids understanding of butterfly-host plant networks. Sci. Rep..

[13] Becerra JX (1997). Insects on plants: Macroevolutionary chemical trends in host use. Science.

[14] Bush GL (1969). Sympatric host race formation and speciation in frugivorous flies of the genus. Evolution (NY).

[15] Bush GL, Smith JJ (1998). The genetics and ecology of sympatric speciation: A case study. Res. Popul. Ecol. (Kyoto).

[16] Linn C, Nojima S, Roelofs W (2005). Antagonist effects of non-host fruit volatiles on discrimination of host fruit by Rhagoletis flies infesting apple (Malus pumila), hawthorn (Crataegus spp), and flowering dogwood (Cornus florida). Entomol Exp Appl..

[17] Linn C (2003). Fruit odor discrimination and sympatric host race formation in *Rhagoletis*. Proc. Natl. Acad. Sci. U. S. A..

[18] Nojima, S., Linn, C., Morris, B., Zhang, A. & Roelofs, W. Identification of host fruit volatiles from hawthorn (*Crataegus* spp.) attractive to hawthorn-origin *Rhagoletis pomonella* flies. *J. Chem. Ecol.* **29**, 321–336 (2003). 10.1023/a:1022677827233 12737261

[19] Nojima S, Linn C, Morris B, Zhang A, Roelofs W (2003). Identification of host fruit volatiles from flowering dogwood (*Cornus florida*) attractive to dogwood-origin *Rhagoletis pomonella* flies. J. Chem. Ecol..

[20] Ohshima I, Yoshizawa K (2006). Multiple host shifts between distantly related plants, Juglandaceae and Ericaceae, in the leaf-mining moth *Acrocercops leucophaea* complex (Lepidoptera: Gracillariidae). Mol. Phylogenet. Evol..

[21] Vanbergen AJ (2003). Host shifting by *Operophtera brumata* into novel environments leads to population differentiation in life-history traits. Ecol. Entomol..

[22] Dethier VG (1982). Mechanism of host-plant recognition. Entomol. Exp. Appl..

[23] Honda K (1995). Chemical basis of differential oviposition by lepidopterous insects. Arch. Insect Biochem. Physiol..

[24] Murphy SM, Feeny P (2006). Chemical facilitation of a naturally occurring host shift by *Papilio machaon* butterflies (Papilionidae). Ecol. Monogr..

[25] Kumata T, Kuroko H, Ermolaev V (1988). Japanese species of the Acrocercops-group (Lepidoptera: Gracillariidae). Part I. Insecta Matsumurana. New Ser..

[26] Ohshima I (2008). Host race formation in the leaf-mining moth *Acrocercops transecta* (Lepidoptera: Gracillariidae). Biol. J. Linn. Soc..

[27] Ohshima I (2012). Genetic mechanisms preventing the fusion of ecotypes even in the face of gene flow. Sci. Rep..

[28] Renwick. Chemical ecology of oviposition in phytophagous insects. *Experientia* **45**, 223–228 (1989).

[29] Visser J (1986). Host odor perception in phytophagous insects. Annu. Rev. Entomol..

[30] Ma WC, Schoonhoven LM (1973). Tarsal contact chemosensory hairs of the large white butterfly *Pieris brassicae* and their possible rôle in oviposition behaviour. Entomol. Exp. Appl..

[31] Du Y-J, Loon JJAV, Renwick JAA (1995). Contact chemoreception of oviposition-stimulating glucosinolates and an oviposition-deterrent cardenolide in two subspecies of *Pieris napi*. Physiol. Entomol..

[32] Städler E, Renwick JAA, Radke CD, Sachdev-Gupta K (1995). Tarsal contact chemoreceptor response to glucosinolates and cardenolides mediating oviposition in *Pieris rapae*. Physiol. Entomol..

[33] Baur R, Haribal M, Renwick JAA, Städler E (1998). Contact chemoreception related to host selection and oviposition behaviour in the monarch butterfly Danaus plexippus. Physiol Entomol.

[34] Eguchi E, Tominaga Y (1999). Atlas of arthropod sensory receptors: Dynamic morphology in relation to function.

[35] Ono H, Nishida R, Kuwahara Y (2000). A dihydroxy-γ-lactone as an oviposition stimulant for the swallowtail butterfly, *Papilio bianor*, from the rutaceous plant. Orixa Japonica. Biosci. Biotechnol. Biochem..

[36] Ohsugi T, Nishida R, Fukami H (1991). Multi-component system of oviposition stimulants for a Rutaceae-feeding swallowtail butterfly, *Papilio xuthus* (Lepidoptera: Papilionidae). Appl. Entomol. Zool..

[37] Ômura, H. *Plant secondary metabolites in host selection of butterfly*. *Chemical Ecology of Insects (ed. by J. Tabata), CRC Press, Boca Raton, Florida* (CRC Press, 2018).

[38] Sakakibara J, Hotta Y, Yasue M (1975). Studies on the constituents of *Lyonia ovalifolia* Drude var. elliptica Hand.-Mazz. XIX. Structure of triterpenoid glucoside, lyofolic acid (3). On the structure of lyofoligenic acid. Chem. Pharm. Bull..

[39] Sakakibara J, Hotta Y, Yasue M (1974). Studies on the constituents of *Lyonia ovalifolia* Drude var. elliptica Hand.-Mazz XVII. Structure of a triterpene arabinoside, ovalifolioside. (2). Chem. Pharm. Bull..

[40] Yasue M, Sakakibara J, Kato T (1971). Studies on the constituents of *Lyonia ovalifolia* Drude var. elliptica Hand.-Mazz. XII. Structure of ovalifolioside, a triterpenoid glycoside. Chem. Pharm. Bull..

[41] Yasue M, Sakakibara J, Kato T, Yazaki H, Hotta Y (1970). Studies on the constituents of *Lyonia ovalifolia* Drude var. elliptica Hand.-Mazz. XI. Structure of triterpenoid glucoside, lyofolic acid (1). Chem. Pharm. Bull..

[42] Yasue M, Kaiya T, Wada A (1967). Studies on the constituents of *Lyonia ovalifolia* Sieb. et Zucc. Var. elliptica Hand.-Mazz. VI. On the triterpenoid and steroid components of the leaves. Chem. Pharm. Bull..

[43] Spindler K (1977). Catalytical oxidation of ecdysteroids to 3-dehydro products and their biological activities. J. Insect Physiol..

[44] Huang X, Renwick JAA (1994). Relative activities of glucosinolates as oviposition stimulants for *Pieris rapae* and *P*. *napi* oleracea. J. Chem. Ecol..

[45] Nishida R (2014). Chemical ecology of insect-plant interactions: Ecological significance of plant secondary metabolites. Biosci. Biotechnol. Biochem..

[46] Stensmyr MC (2009). Drosophila sechellia as a model in chemosensory neuroecology. Ann. NY Acad. Sci..

[47] Dekker T, Ibba I, Siju KP, Stensmyr MC, Hansson BS (2006). Olfactory shifts parallel superspecialism for toxic fruit in *Drosophila melanogaster* sibling. D. Sechellia. Curr. Biol..

[48] Stensmyr MC, Dekker T, Hansson BS (2003). Evolution of the olfactory code in the *Drosophila melanogaster* subgroup. Proc. R. Soc. B Biol. Sci..

[49] McBride CS, Arguello JR (2007). Five *Drosophila* genomes reveal nonneutral evolution and the signature of host specialization in the chemoreceptor superfamily. Genetics.

[50] Matsuo T, Sugaya S, Yasukawa J, Aigaki T, Fuyama Y (2007). Odorant-binding proteins OBP57d and OBP57e affect taste perception and host-plant preference in *Drosophila sechellia*. PLoS Biol..

[51] Ryuda M (2013). Gustatory sensing mechanism coding for multiple oviposition stimulants in the swallowtail butterfly. Papilio xuthus. J. Neurosci..

[52] Matsubayashi KW, Ohshima I, Nosil P (2010). Ecological speciation in phytophagous insects. Entomol. Exp. Appl..

[53] Thompson JN (1988). Evolutionary ecology of the relationship between oviposition preference and performance of offspring in phytophagous insects. Entomol. Exp. Appl..

[54] Thompson JN, Pellmyr O (1991). Evolution of oviposition behavior and host preference in Lepidoptera. Annu. Rev. Entomol..

[55] Larsson S, Ekbom B (1995). Oviposition mistakes in herbivorous insects: confusion or a step towards a new host plant?. Oikos.

[56] Ohshima I (2005). Techniques for continuous rearing and assessing host preference of a multivoltine leaf-mining moth, *Acrocercops transecta* (Lepidoptera: Gracillariidae). Entomol. Sci..

