# Supplementary material for: Oviposition stimulants underlying different preferences between host races in the leaf-mining moth Acrocercops transecta (Lepidoptera: Gracillariidae)
Source: Sci Rep. 2022 Aug 25;12:14498. doi: 10.1038/s41598-022-18238-0 (PMC9411557; doi:10.1038/s41598-022-18238-0)
Supplement: Supplementary file 2 — Supplementary Information 1. [file 41598_2022_18238_MOESM2_ESM.pdf]

Supplementary Information

Oviposition stimulants underlying different preferences between host races in the leaf-mining moth *Acrocercops transecta* (Lepidoptera: Gracillariidae)

Tomoko Katte[1,5], Shota Shimoda[1,5], Takuya Kobayashi[1,5], Ayako Wada-Katsumata[2], Ritsuo Nishida[1], Issei Ohshima[3,4], Hajime Ono[1,5*]

[1]Graduate School of Agriculture, Kyoto University, Kyoto 606-8502, Japan.

[2]Department of Entomology and Plant Pathology, and W.M. Keck Center for Behavioral Biology, North Carolina State University, NC 27695-7613, USA.

[3]Department of Life and Environmental Sciences, Kyoto Prefectural University, 606-8522, Kyoto, Japan. [4]Center for Frontier Natural History, Kyoto Prefectural University, 606-8522, Kyoto, Japan. [5]These authors contributed equally to this study. *email: ono.hajime.5a@kyoto-u.ac.jp

Contents:

Document

Identification of the triterpenoid glycosides, **1** and **3**

Table S1 The numbers of oviposited eggs in ablation experiments (Fig. 1A)

Table S2 The numbers of oviposited eggs of the *Lyonia* race in four-choice assays (Fig. 2A)

Table S3 The numbers of oviposited eggs of the *Juglans* race in four-choice assays (Fig. 2B)

Table S4 The numbers of oviposited eggs of the *Lyonia* race in four-choice assays (Fig.3B)

Table S5 The numbers of oviposited eggs of the *Lyonia* race in four-choice assays (Fig. 3C)

Table S6 The numbers of oviposited eggs in two-choice assays (Fig. 3E)

Table S7 The numbers of oviposited eggs in two-choice assays (Fig. 3F)

Table S8 The numbers of oviposited eggs of the *Juglans* race in two-choice assays

Table S9. ^{13}C NMR data of compounds **1**–**3** in C_5D_5N

Table S10. The numbers of oviposited eggs in two-choice assays (Fig. 5A)

Figure

Figure S1. Comparison of ^{1}H NMR spectra. (A) Oxidized product of **1** (lyofolic acid). (B) **2** (24-dehydrolyofolic acid) isolated from *Lyonia ovalifolia* leaves.

NMR spectra (Figure S2-S28)

Figure S2. ^{1}H NMR spectrum of **1**.

Figure S3. ^{13}C NMR spectrum of **1**.

Figure S4. ^{13}C NMR spectrum of **1** (enlarged figure).

Figure S5. ^{1}H-^{1}H COSY spectrum of **1**.

Figure S6. ^{1}H-^{1}H COSY spectrum of **1** (enlarged figure).

Figure S7. HSQC spectrum of **1**.

Figure S8. HSQC spectrum of **1** (enlarged figure).

Figure S9. HMBC spectrum of **1**.

Figure S10. HMBC spectrum of **1** (enlarged figure).

Figure S11. ^{1}H NMR spectrum of **2**.

Figure S12. ^{13}C NMR spectrum of **2**.

Figure S13. ^{13}C NMR spectrum of **2** (enlarged figure).

Figure S14. ^{1}H-^{1}H COSY spectrum of **2**.

Figure S15. ^{1}H-^{1}H COSY spectrum of **2** (enlarged figure).

Figure S16. Edited-HSQC spectrum of **2**. Methine and methyl resonances are represented by black contours, and methylene resonances are defined by red contours.

Figure S17. Edited-HSQC spectrum of **2** (enlarged figure).

Figure S18. HMBC spectrum of **2**.

Figure S19. HMBC spectrum of **2** (enlarged figure).

Figure S20. ^{1}H NMR spectrum of **3**.

Figure S21. ^{13}C NMR spectrum of **3**.

Figure S22. ^{13}C NMR spectrum of **3** (enlarged figure).

Figure S23. ^{1}H-^{1}H COSY spectrum of **3**.

Figure S24. ^{1}H-^{1}H COSY spectrum of **3** (enlarged figure).

Figure S25. HSQC spectrum of **3**.

Figure S26. HSQC spectrum of **3** (enlarged figure).

Figure S27. HMBC spectrum of **3**.

Figure S28. HMBC spectrum of **3** (enlarged figure).

Movie S1. Egg-laying behavior by a female of *A. transecta* by tapping a leaf with its antennae.

Identification of the triterpenoid glycosides, 1 and 3

The molecular formula of **1** was determined to be $C_{38}H_{62}O_{11}$ by HR-ESI-MS analysis. The ^{1}H-NMR spectrum of **1** revealed two doublet signals of methine protons (δ 0.61 and 0.39, J = 3.5 Hz) at a cyclopropane ring and seven methyl protons (δ 2.00, 1.54, 1.51, 1.24, 1.20, 1.05 and 1.01), which suggests that **1** is a cycloartane-type triterpene. The multiple signals of protons (δ 4.96-4.81 and 4.24-3.73) adjacent oxygens including an anomeric proton (δ 4.82) indicate that **1** contains a sugar moiety. The ^{1}H-^{1}H COSY spectrum revealed a connectivity of five methine protons and one methylene protons, indicating the presence of a hexose moiety. This hexose was identified as glucose by a sequential trans-diaxial relationship of the H-1'/H-2'/H-3'/H-4'/H-5' and two correlated methylene signals (H-6'a, b). The ^{13}C-NMR spectrum of **1** revealed two signals of carbons adjacent carbonyl group (δ 178.9 and 170.8), a signal of an anomeric carbon (δ 102.4), and 9 signals of carbons adjacent oxygens or carbonyl group (δ 83.3, 79.1, 78.6, 75.1, 75.0, 72.7, 71.8, 64.8 and 62.9). The ^{13}C-NMR spectrum of **1** was very similar to that of lyofolic acid, as previously reported (Table S9) (Sakakibara et al., 1975). Furthermore, the assignment of proton and carbon signals by ^{1}H-^{1}H COSY, HSQC and HMBC analyses supported the structure of **1** as lyofolic acid. Because the optical rotation of **1**, $[\alpha]_D^{25}$ –18.8 (c = 1.0, CH_3OH), was in agreement with that reported ($[\alpha]_D^{15}$ –18.8 , CH_3OH) (Sakakibara et al., 1967), **1** was concluded to be lyofolic acid.

The molecular formula of **3** was determined to be $C_{35}H_{58}O_7$ by HR-ESI-MS analysis. The ^{1}H-NMR spectrum of **3** revealed a signal of an olefinic proton (δ 5.38), an anomeric proton (δ 5.00), multiple signals of protons (δ4.45-3.69) adjacent oxygens, and 7 signals of methyl protons (δ 1.38, 1.26, 1.15, 1.02, 0.94, 0.90 and 0.88). The ^{13}C-NMR spectrum of **3** revealed two signals of olefinic carbons (δ 145.3 and δ 130.7), a

signal of an anomeric carbon (δ 106.5), and seven signals of carbons adjacent oxygens (δ 80.6, 80.4, 74.6, 73.0, 69.8, 66.9 and 64.5). The ^{1}H-^{1}H COSY spectrum revealed a connectivity of four methine protons and one methylene protons, indicating the presence of a pentose moiety. This pentose was identified as arabinose by coupling patterns of ^{1}H-NMR, i.e., *trans*-diaxial relationship of the H-1'/H-2' and H-2'/H-3', and axial-equatorial relationship of the H-3'/H-4'. The ^{1}H-^{1}H COSY, HSQC and HMBC spectra suggests that **3** is an oleanane-type triterpene. The ^{13}C-NMR spectrum of **3** closely correlated with that reported for ovalifoliogenin that is an aglycone of ovalifolioside, with minor differences due to the presence or absence of the arabinose moiety and the different solvents used (Table S9) (Liu et al., 2019). The HMBC spectrum exhibited the correlations between H-1' of arabinose and C-3 of the aglycone. Because the optical rotation of **3**, $[\alpha]_D^{24}$ +54.0 (*c* = 1.0, CH_3OH), was in good agreement with that reported ($[\alpha]_D^{23}$ +69.1, CH_3OH) (Sakakibara et al., 1971), **3** was concluded to be ovalifolioside.

Table S1. The numbers of oviposited eggs in ablation experiments (Fig. 1A)

		Ablation		
A	A (one)	FT	A-FT	None
0	2	3	0	10
0	17	10	0	8
0	17	0	0	12
0	11	9	0	8
0	11	22	0	19
0	43	12	0	2
0	4	2	0	6
21	19	1	0	10
0	11	1	1	5
16	1	6	0	4
0		11	0	0
6		20	0	0
0		11	0	7
0		14	0	5
1		0	1	3
0		17	0	18
1		4	0	14
			0	8
			0	

Table S2. The numbers of oviposited eggs of the *Lyonia* race in four-choice assays (Fig. 2A)

EtOAc	Ether	Hexane	Control
6	0	0	0
13	2	0	0
0	1	0	0
0	1	0	0
0	5	0	0
4	6	0	0
0	0	0	0
6	2	0	0
1	0	0	0
2	1	0	0
1	0	0	0
7	2	0	0
2	1	0	0
4	3	0	0
26	27	0	0
18	3	0	2
0	2	0	0
0	0	0	0
0	0	0	0
0	0	0	0
5	3	0	0

Table S3. The numbers of oviposited eggs of the *Juglans* race in four-choice assays (Fig. 2B)

EtOAc	Ether	Hexane	Control
0	0	0	0
0	0	0	0
0	0	0	0
0	0	0	0
0	0	0	0
0	0	0	0
0	0	0	0
0	0	0	0
0	0	0	0
0	0	0	0
23	0	0	0
0	0	0	0
0	0	0	0
0	0	0	0
0	0	0	0
0	0	0	0
0	0	0	0
0	0	0	0
0	0	0	0
0	0	0	0
0	0	0	0
0	0	0	0
0	0	0	0
0	0	0	0

Table S4. The numbers of oviposited eggs of the *Lyonia* race in four-choice assays (Fig. 3B)

Fr. EA	Fr. AC	Fr. EO	Control
0	0	0	0
0	0	0	0
0	1	0	0
0	0	1	0
0	0	0	0
0	0	0	0
0	0	0	0
0	0	0	0
0	13	0	1
0	8	0	0
0	0	0	0
0	0	0	0
0	0	0	0
0	0	0	0
0	0	0	0
1	1	0	0
0	0	0	0
2	5	1	0
0	3	3	0
1	34	0	0
0	13	0	0
0	1	0	0
0	1	1	3
0	0	0	0
0	0	0	0
0	8	0	0
0	5	0	0
0	0	2	0
0	7	0	1
0	13	0	4
0	0	0	0
0	0	0	0

Table S5. The numbers of oviposited eggs of the *Lyonia* race in four-choice assays (Fig. 3C)

Fr. 1-3	Fr. 4-6	Control 1	Control 2
0	1	0	0
0	1	1	0
0	0	0	0
0	0	0	0
0	0	0	0
0	0	0	0
0	0	0	1
0	0	0	0
0	9	0	0
0	5	1	0
0	0	0	0
0	0	0	0
0	0	0	0
0	0	0	0
0	0	0	0
0	1	0	0
0	0	0	0
0	0	0	0
0	0	0	0
0	0	0	0

Table S6. The numbers of oviposited eggs in two-choice assays (Fig. 3E)

Fr. A		Fr. 4-6 + Fr. A		Fr. 4 + Fr. A		Fr. 5 + Fr. A		Fr. 6 + Fr. A		Fr. 6	
Sample	Control	Sample	Control	Sample	Control	Sample	Control	Sample	Control	Sample	Control
1	0	0	0	0	0	0	0	5	0	0	0
0	0	3	0	0	0	0	0	0	0	0	0
0	0	0	0	0	0	0	0	0	0	0	0
0	0	0	0	0	0	1	0	0	0	0	0
0	0	4	0	0	0	0	0	1	0	0	0
0	0	6	0	0	0	0	0	14	0	0	0
0	0	4	0	6	0	0	0	5	0	0	0
0	0	15	0	1	0	0	0	0	0	0	0
0	0	0	0	0	0	0	0	0	0	0	0
0	0	0	0	0	0	0	0	0	0	0	0
0	0	0	0	1	0	0	0	1	0	0	0
0	0	0	0	0	0	0	0	0	0	1	0
0	0	0	0	0	0	0	0	0	0	0	0
0	0	0	0	0	0	1	0	3	0	0	0
0	0	0	0	0	0	0	0	0	0	0	0
0	0	0	0	0	0	0	0	0	0	0	0
0	0	0	0	0	0	0	0	3	1	0	0
0	0	1	0	0	0	0	0	0	0	2	0
0	0			0	0			0	0	0	0
0	0			0	0			0	0	0	0
0	0							0	0	0	0
								0	0	0	0
								0	0	0	0
										0	0
										0	0
										0	0
										0	0
										0	0
										0	0
										1	0
										0	0
										0	0

Table S7. The numbers of oviposited eggs in two-choice assays (Fig. 3F)

Fr. A		**1** (LA)		**2** (Dh-LA)		**1** + Fr. A		**2** + Fr. A		**1** + **2** + Fr. A		**3** (OF) + Fr. A	
Sample	Control	Sample	Control	Sample	Control	Sample	Control	Sample	Control	Sample	Control	Sample	Control
1	0	0	0	0	0	0	0	0	0	1	0	0	0
0	0	0	0	0	0	0	0	0	0	0	0	0	0
0	0	0	0	0	0	0	0	0	0	0	0	0	0
0	0	0	0	0	0	0	0	0	0	1	0	0	0
0	0	0	0	0	0	0	0	3	1	0	0	0	0
0	0	0	0	0	0	0	0	1	0	0	0	0	0
0	0	0	0	0	0	0	0	0	0	0	0	0	0
0	0	0	0	0	0	0	0	0	0	0	0	0	0
0	0	0	0	0	0	0	0	0	0	0	0	0	0
0	0	0	0	0	0	4	0	0	0	0	0	0	0
0	0	0	0	0	0	0	0	0	0	0	0	0	0
0	0	0	0	0	0	0	0	0	0	6	0	1	0
0	0	0	0	0	0	0	0	10	0	0	0	0	1
0	0	0	0	0	0	0	0	6	0	0	0	0	0
0	0	0	0	0	0	0	0	4	0	0	0	0	0
0	0	0	0	0	0	0	0	2	1	0	0	0	0
0	0	0	0	0	0	0	0	0	0	4	0	0	0
0	0	0	0	0	0	5	0	0	0	0	0	0	0
0	0	0	0	0	0	0	0	1	0	7	0	0	0
0	0	0	0	0	0	0	0	0	0	0	0		
0	0	0	0	0	0	0	0	0	0				
		0	0			0	0	0	0				
		0	0			0	0	0	0				
		0	0			2	0	0	0				
		0	0			0	0	5	0				
						5	0	7	0				
						0	0	0	0				
						3	0	0	0				
						0	0						
						0	0						
						0	0						
						0	0						

4	0
1	0

Table S8. The numbers of oviposited eggs of the *Juglans* race in two-choice assays

**1** (LA) + Fr. A	
Sample	Control
0	0
0	0
0	0
0	0
0	0
0	0
0	0
0	0
0	0
0	0
0	0
0	0
0	0
0	0
0	0
0	0
0	0
0	0
0	0
0	0
0	0
0	0
0	0
0	0

Table S9. ^{13}C NMR data of compounds **1**–**3** in C_5D_5N

Position	**1**	**2**	**3**
1	25.2	25.2	79.6
2	28.5	28.5	37.4
3	83.3	83.2	79.9
4	39.5	39.5	43.6
5	41.7	41.7	45.5
6	21.3	21.3	18.0
7	27.3	27.3	33.0
8	47.5	47.5	40.9
9	20.2	20.1	49.1
10	28.1	28.1	43.2
11	28.2	28.2	27.9
12	34.4	34.3	123.8
13	47.7	47.7	144.1
14	62.9	62.9	42.0
15	32.5	32.5	26.7
16	30.2	30.0	27.3
17	53.2	52.8	32.8
18	18.9	18.8	47.6
19	31.0	31.0	47.0
20	36.0	35.8	31.2
21	18.7	18.5	35.0
22	34.1	30.6	37.5
23	29.0	33.4	64.6
24	79.1	216.2	13.5
25	72.7	76.8	12.7
26	26.2*	27.3*	17.6
27	25.8*	27.2*	26.2
28	22.0	22.0	28.6
29	26.8	26.8	33.5
30	178.9	178.8	23.8
1'	102.4	102.4	106.8
2'	75.1*	75.0*	73.1
3'	78.6	78.6	74.7

4'	71.8	71.8	69.6
5'	75.0*	75.0*	66.9
6'	64.8	64.8	
CH3-<u>C</u>O-	170.8	170.7	
<u>C</u>H3-CO-	20.8	20.8	

*Assignments may be interchangeable.

Table S10. The numbers of oviposited eggs in two-choice assays (Fig. 5A)

**1** (LA)		**2** (Oxo-LA)		**3** (OF)		EtOH	
Sample	Control	Sample	Control	Sample	Control	Sample	Control
1	0	0	0	0	0	0	0
0	0	0	0	0	0	0	0
0	0	0	0	0	0	0	0
2	0	0	1	0	0	0	0
0	0	0	0	0	0	0	0
0	0	0	0	0	0	0	0
0	0	1	0	1	3	0	0
1	0	3	2	0	0	0	0
0	0	0	0	0	0	0	0
0	0	0	0	0	0	0	0
0	0	0	0	0	0	0	0
0	0	0	0	0	0	0	0
0	0	0	0	0	0	0	0
0	0	0	0	0	0	0	0
1	0	3	0	0	0	0	0
0	0	0	0	0	0	0	0
0	0	1	0	0	0	0	0
0	0	0	0	0	0	0	0
1	0	6	0	0	0	0	0
1	0	0	0	0	0	0	0
0	0	4	0	0	0	0	0
0	0	0	0	0	0	0	0
0	0	0	0	0	0	0	0
						0	0
						0	0
						0	0
						0	0
						0	0
						0	0

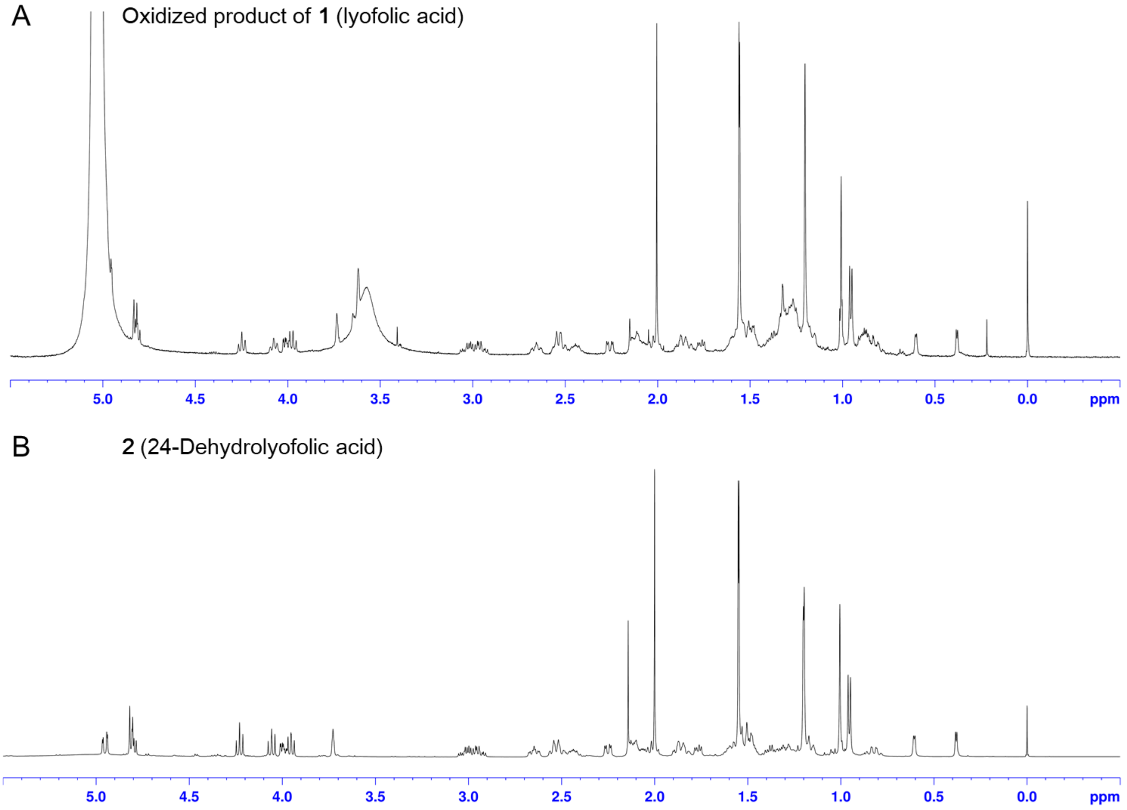

Figure S1. Comparison of ^{1}H NMR spectra. (A) Oxidized product of **1** (lyofolic acid). (B) **2** (24-dehydrolyofolic acid).

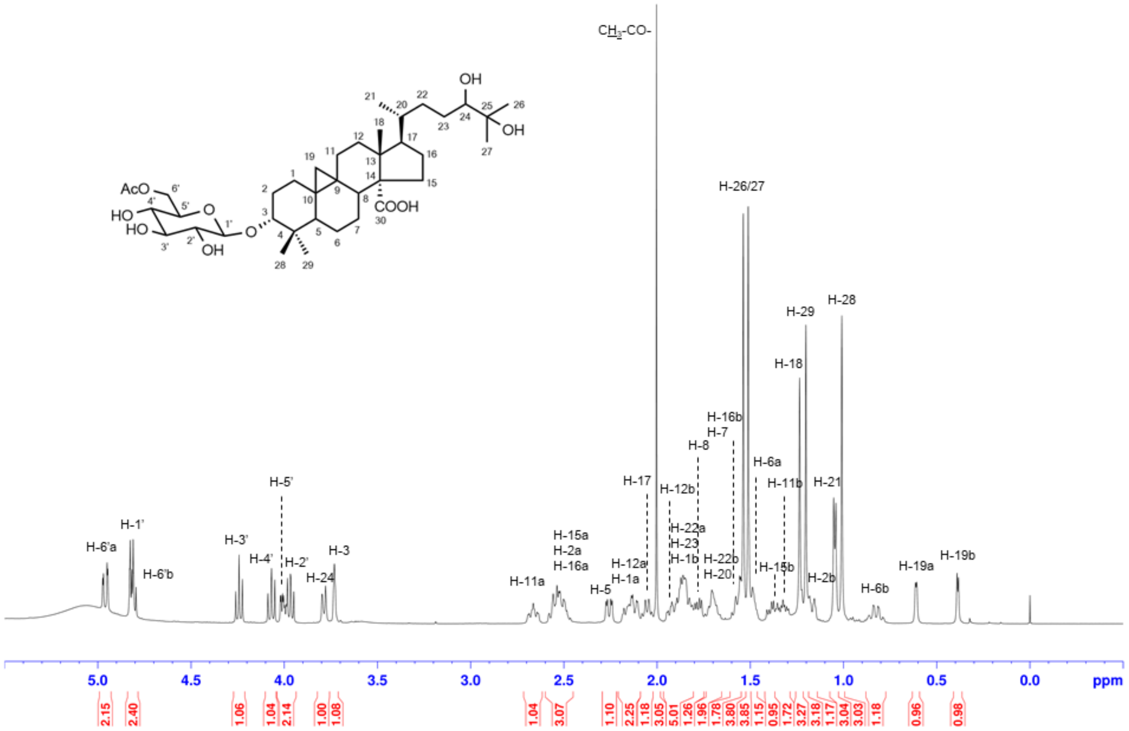

Figure S2. ^{1}H NMR spectrum of **1**.

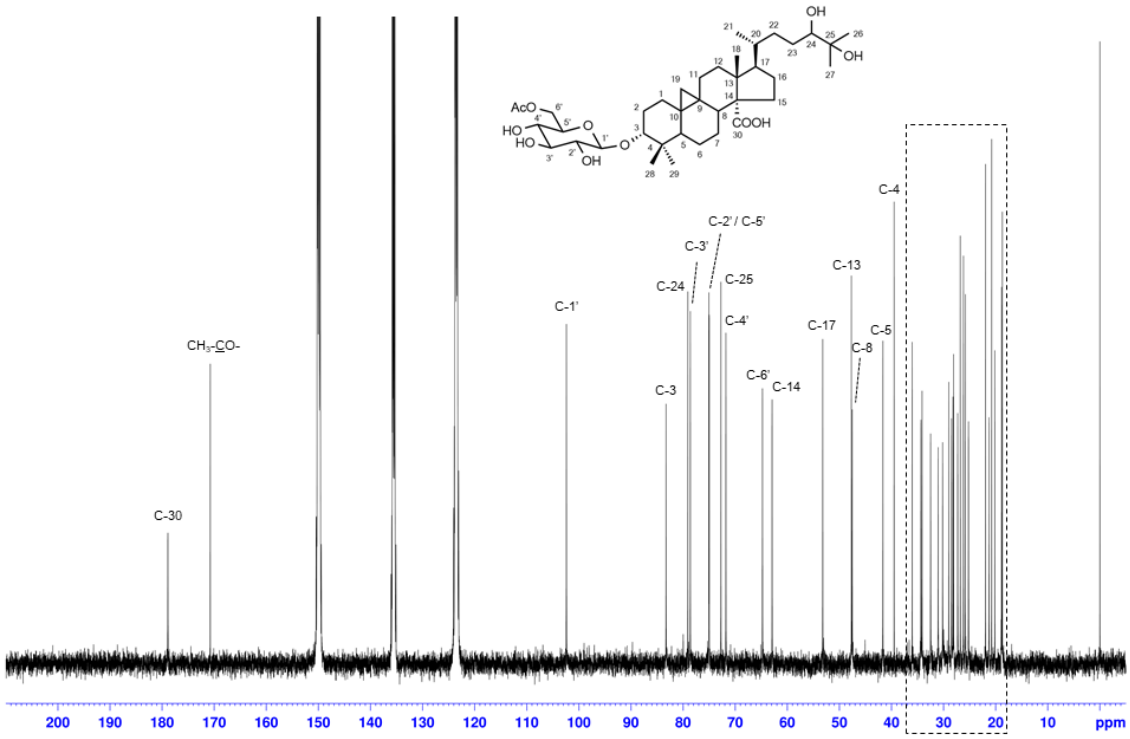

Figure S3. ^{13}C NMR spectrum of **1**.

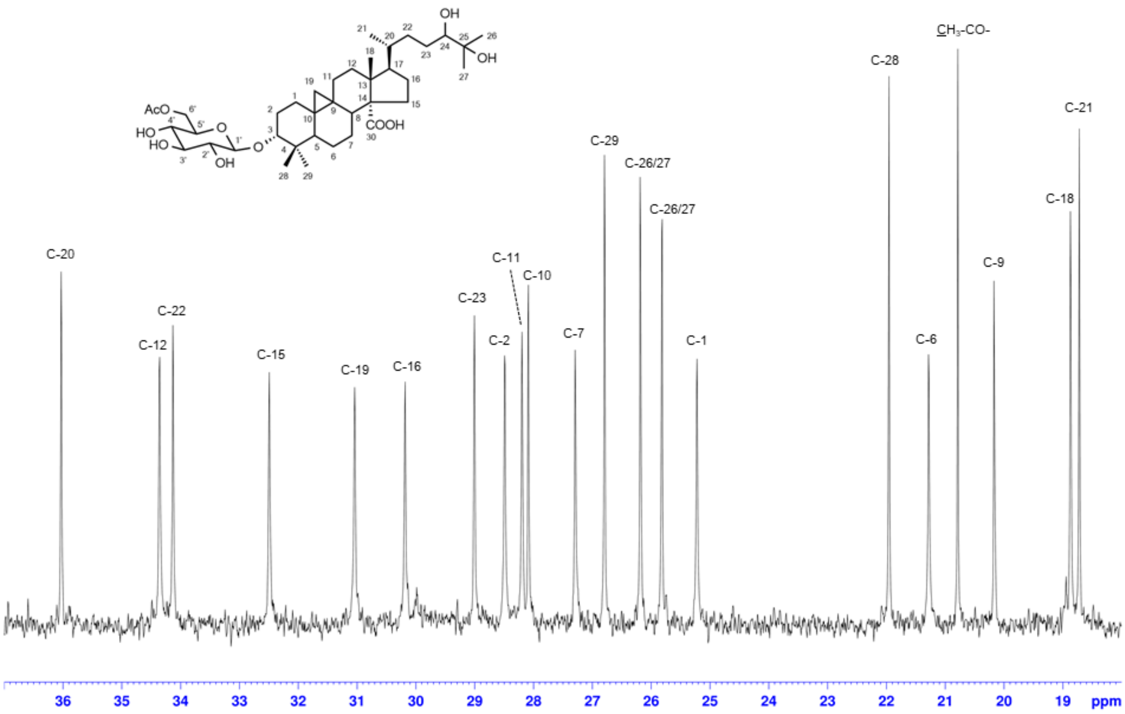

Figure S4. ^{13}C NMR spectrum of **1** (enlarged figure).

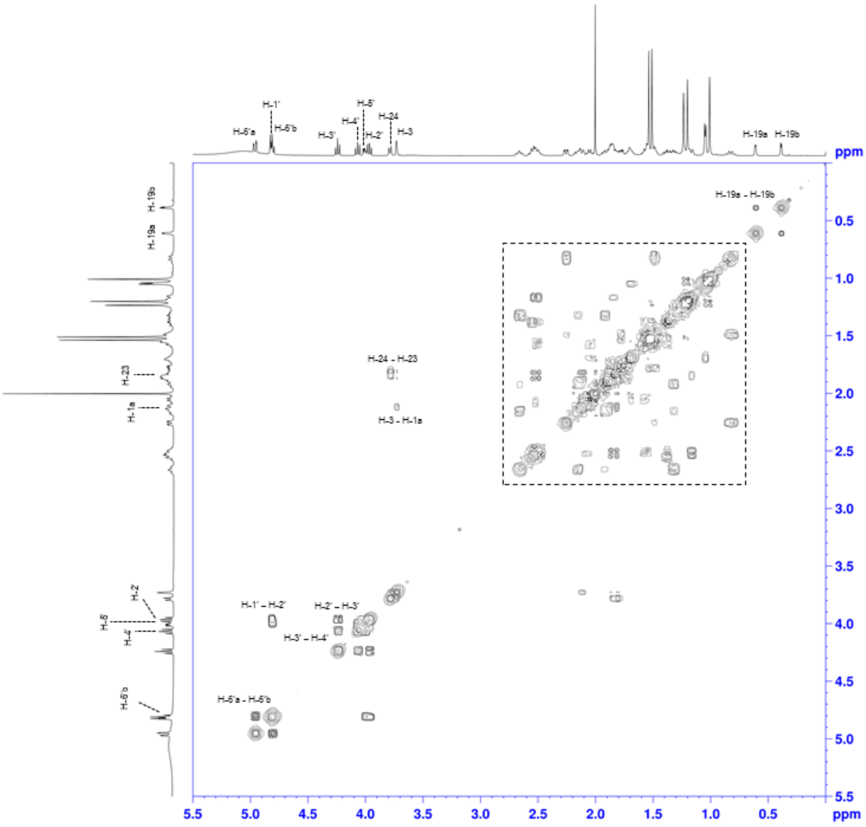

Figure S5. ^{1}H-^{1}H COSY spectrum of **1**.

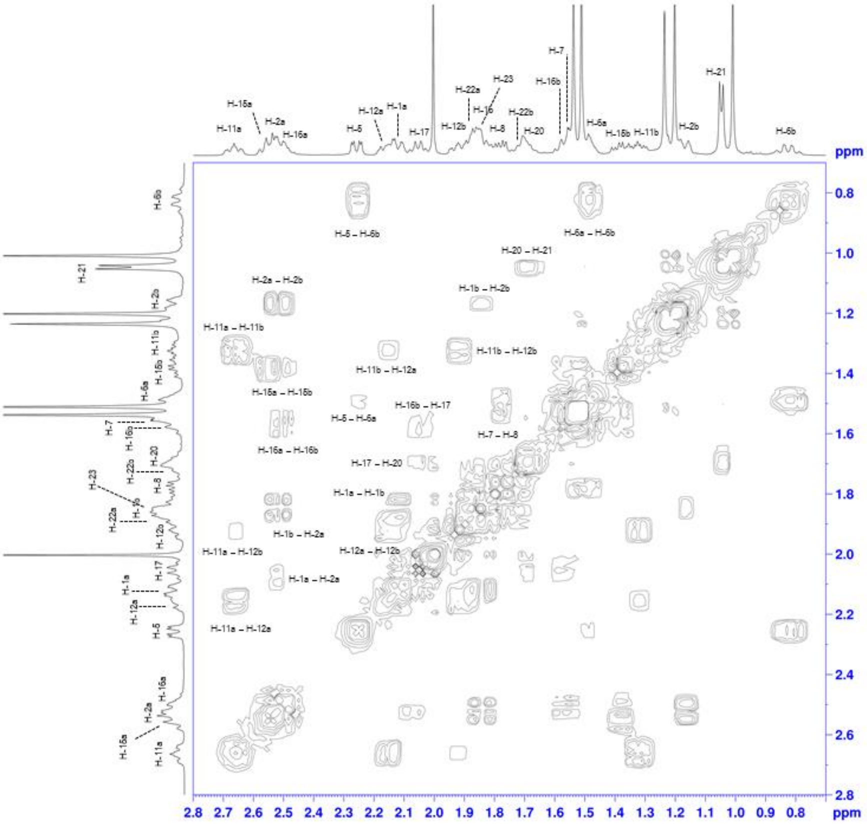

Figure S6. ^{1}H-^{1}H COSY spectrum of **1** (enlarged figure).

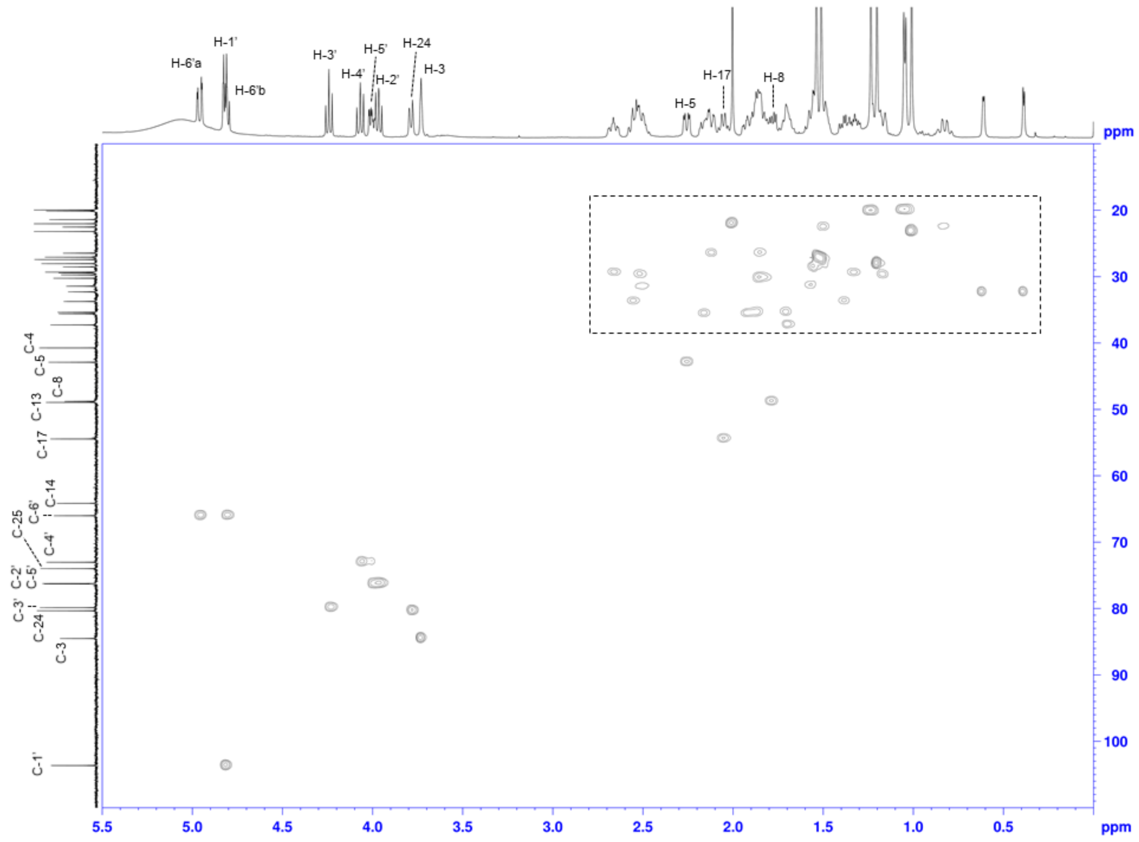

Figure S7. HSQC spectrum of **1**.

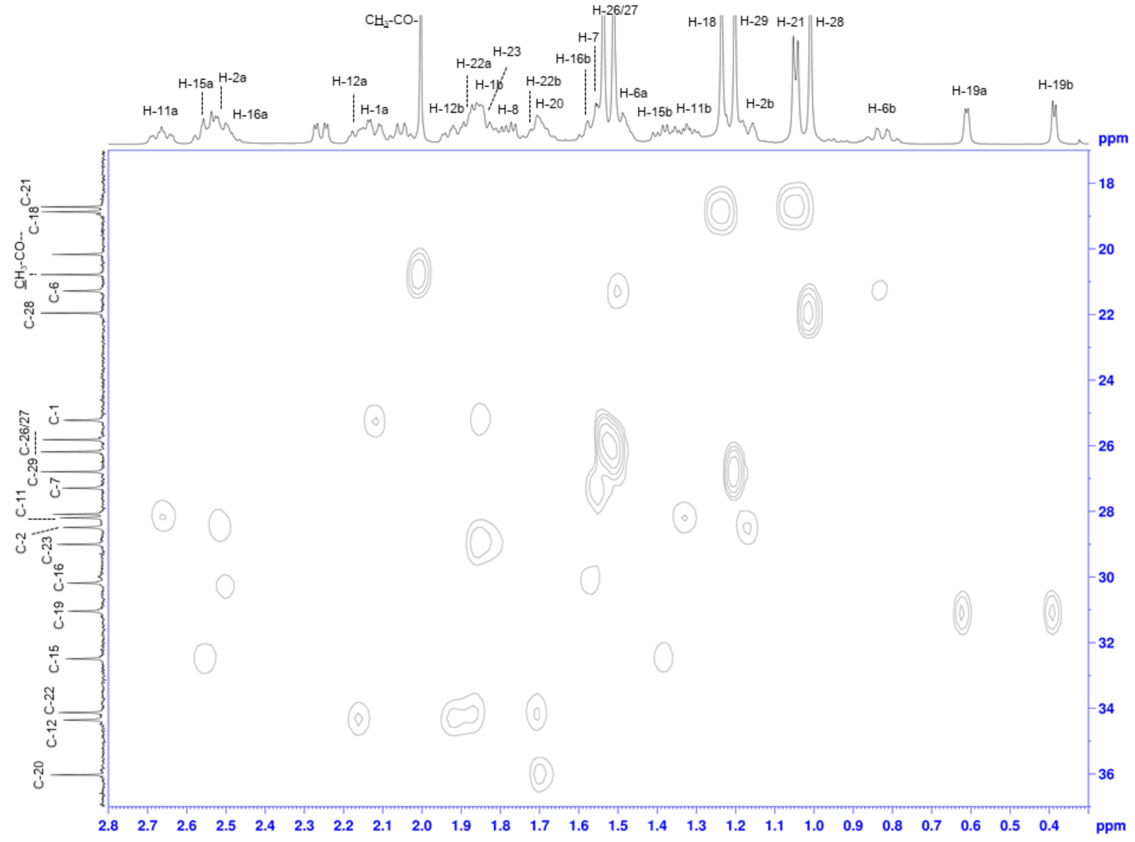

Figure S8. HSQC spectrum of **1** (enlarged figure).

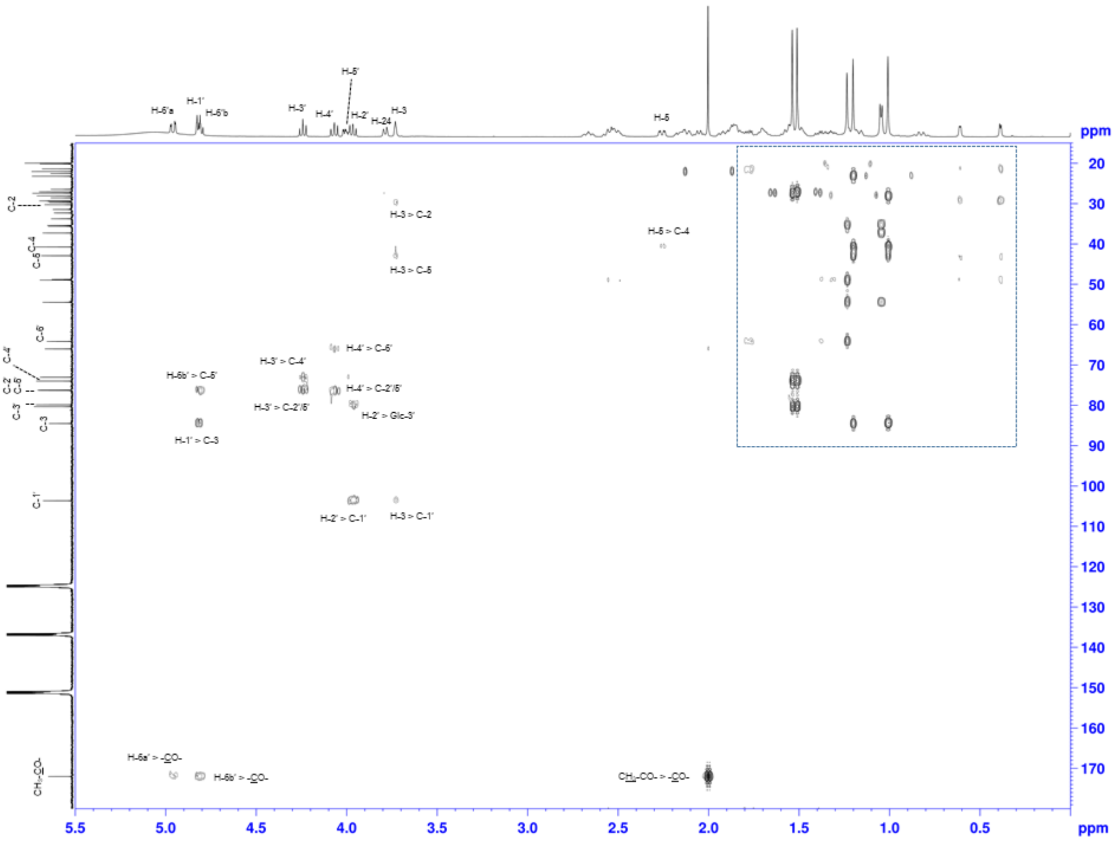

Figure S9. HMBC spectrum of **1**.

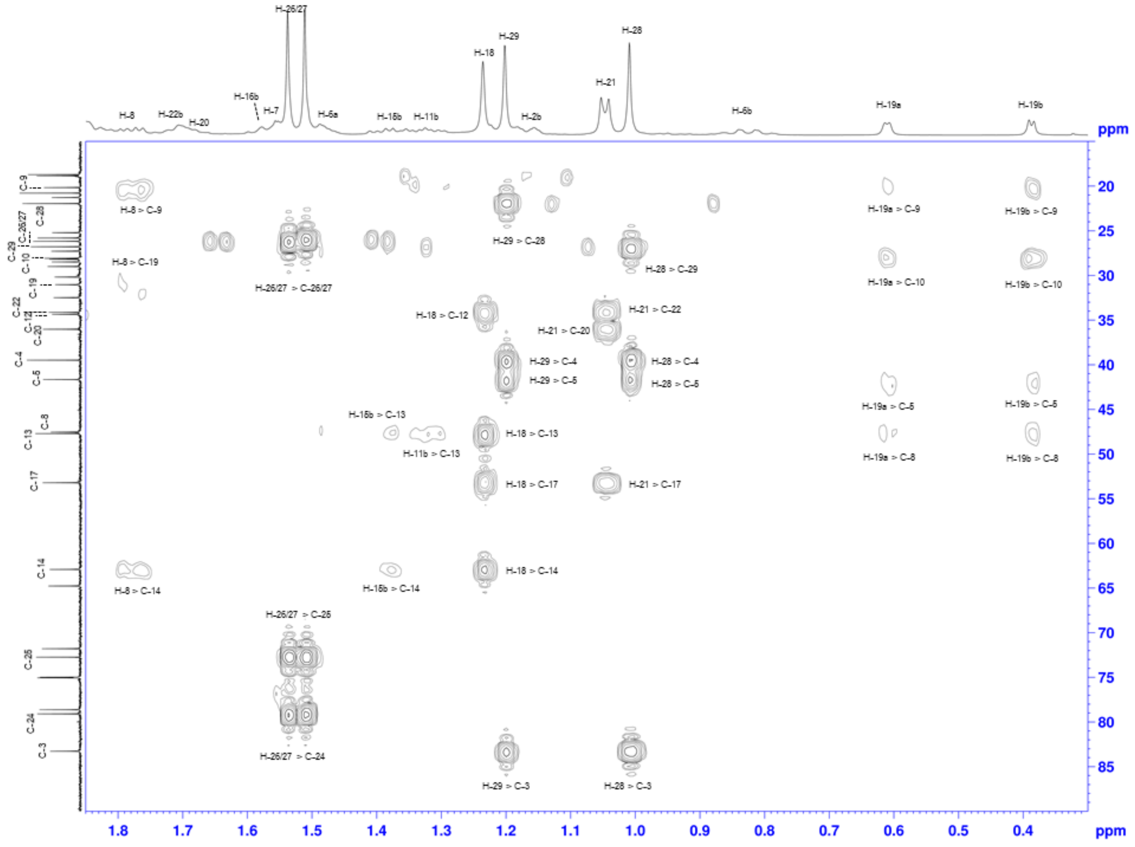

Figure S10. HMBC spectrum of **1** (enlarged figure).

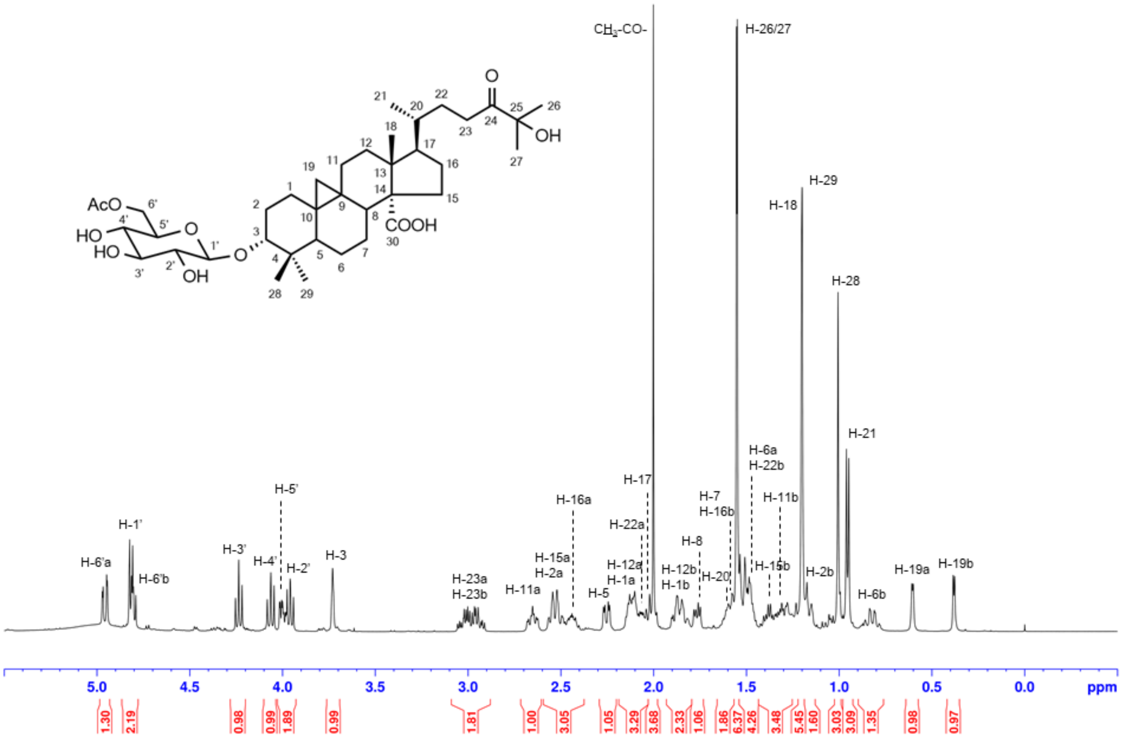

Figure S11. ^{1}H NMR spectrum of **2**.

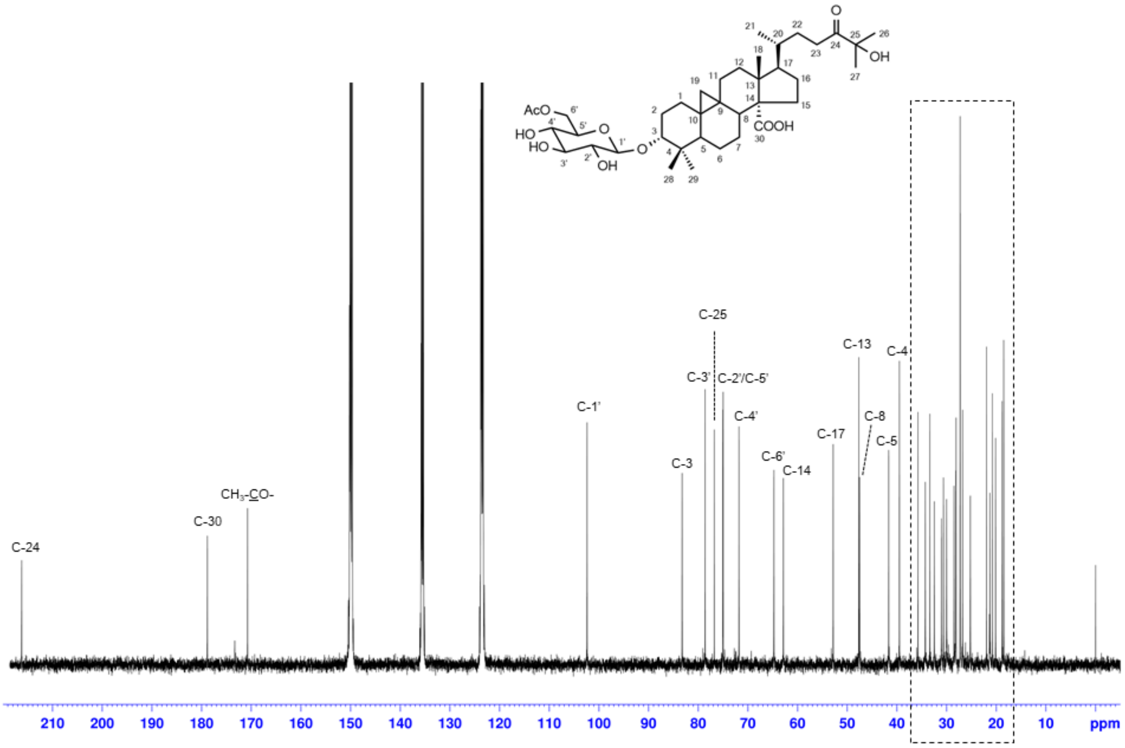

Figure S12. ^{13}C NMR spectrum of **2**.

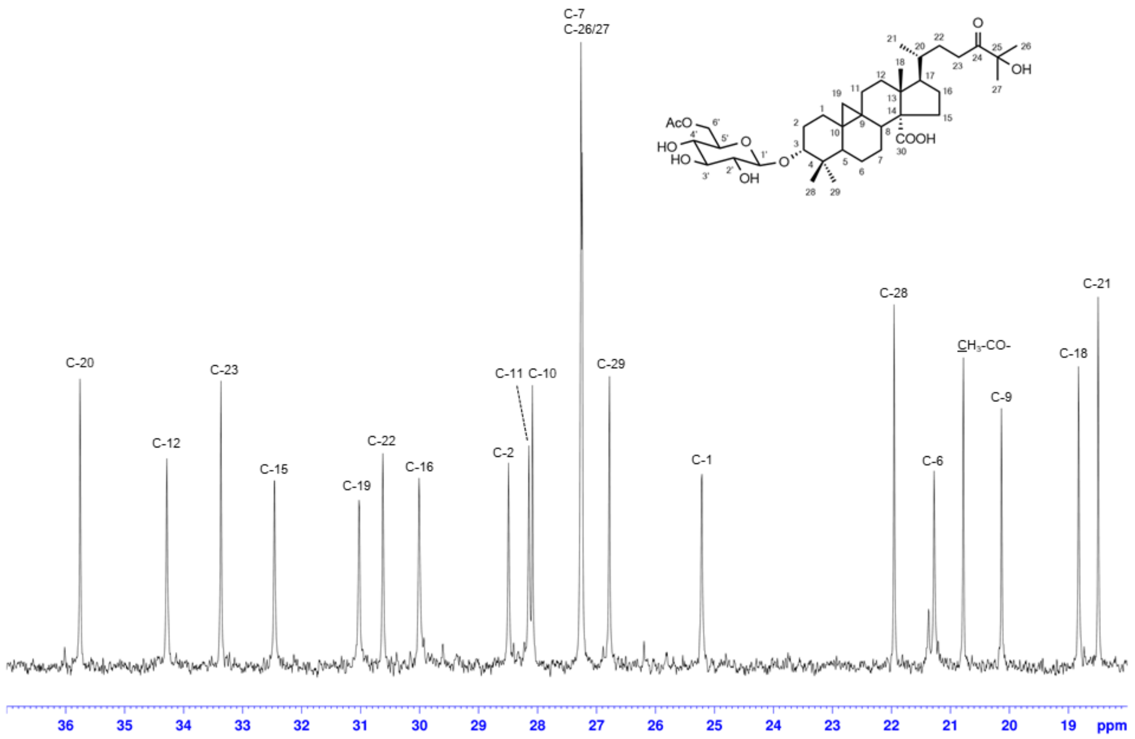

Figure S13. ^{13}C NMR spectrum of **2** (enlarged figure).

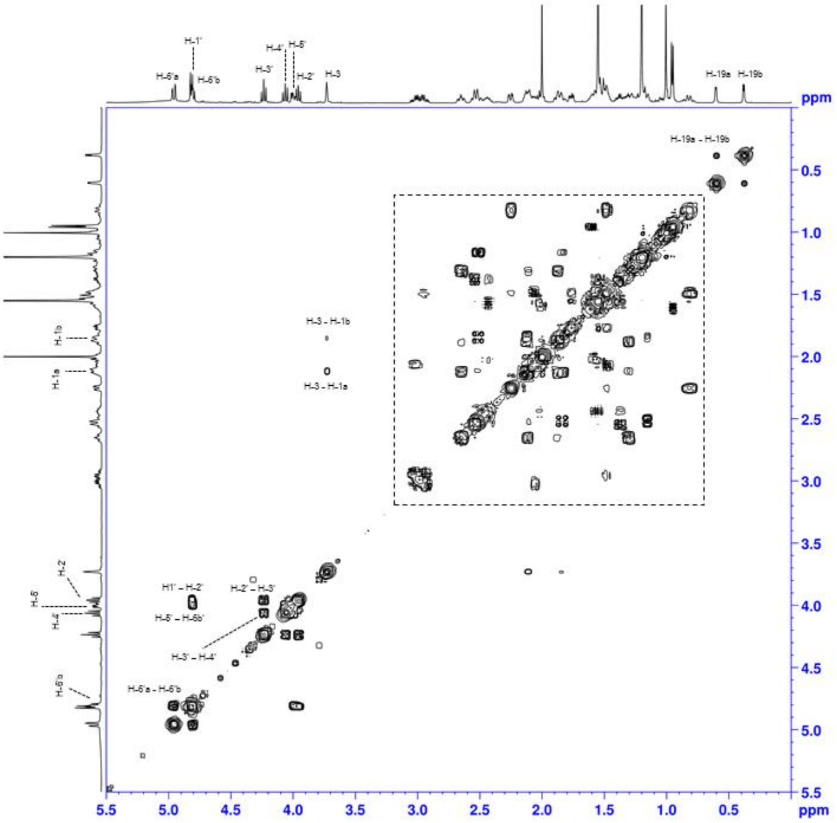

Figure S14. ^{1}H-^{1}H COSY spectrum of **2**.

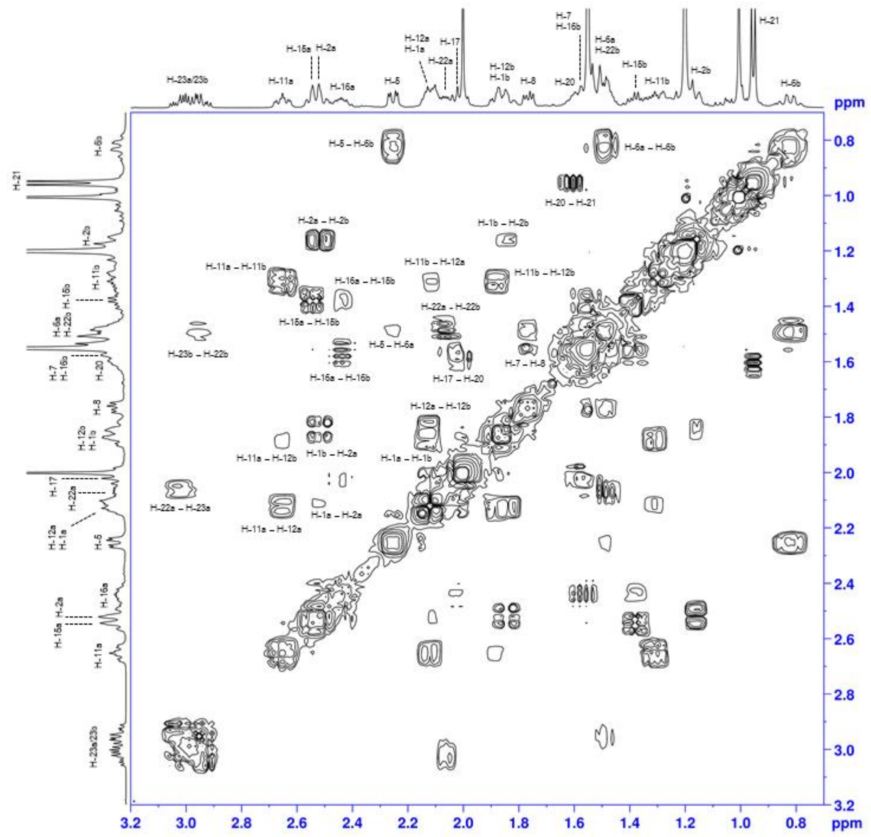

Figure S15. ^{1}H-^{1}H COSY spectrum of **2** (enlarged figure).

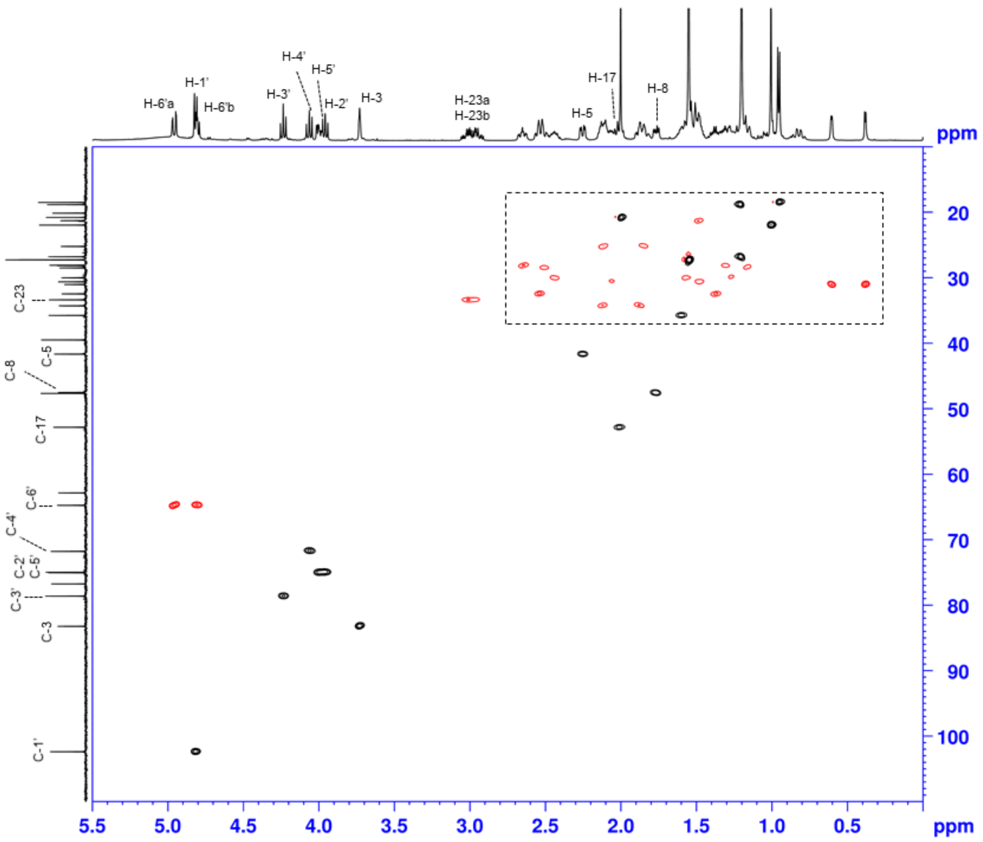

Figure S16. Edited-HSQC spectrum of **2**. Methine and methyl resonances are represented by black contours, and methylene resonances are defined by red contours.

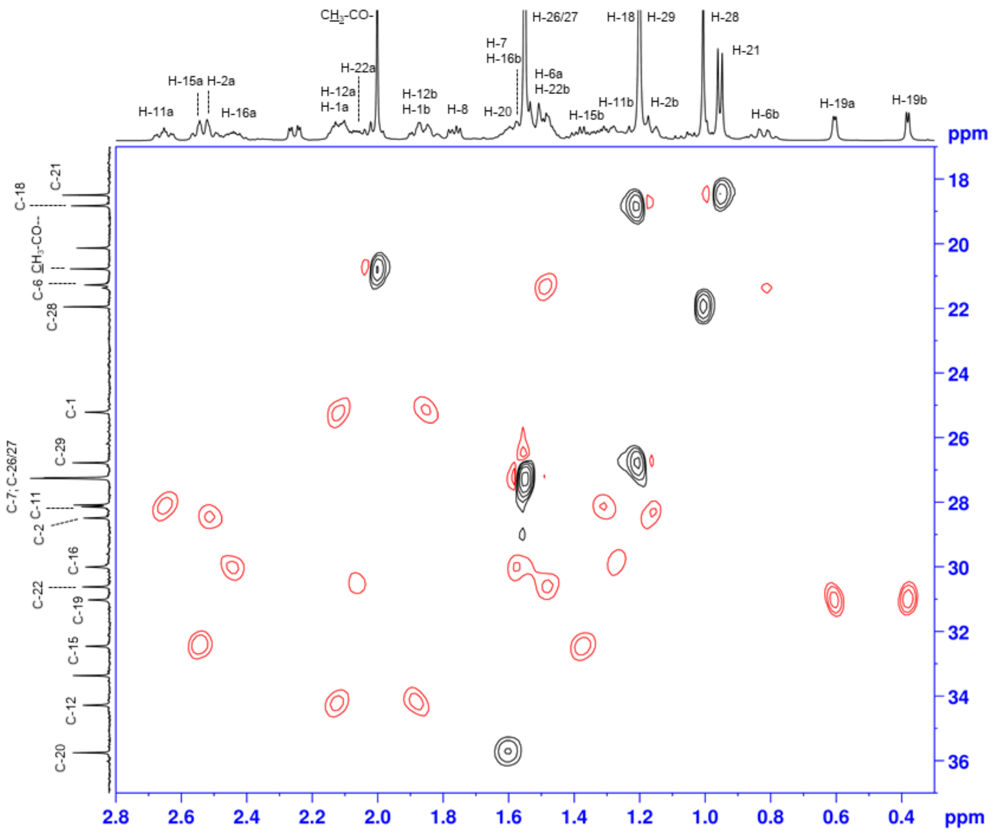

Figure S17. Edited-HSQC spectrum of **2** (enlarged figure).

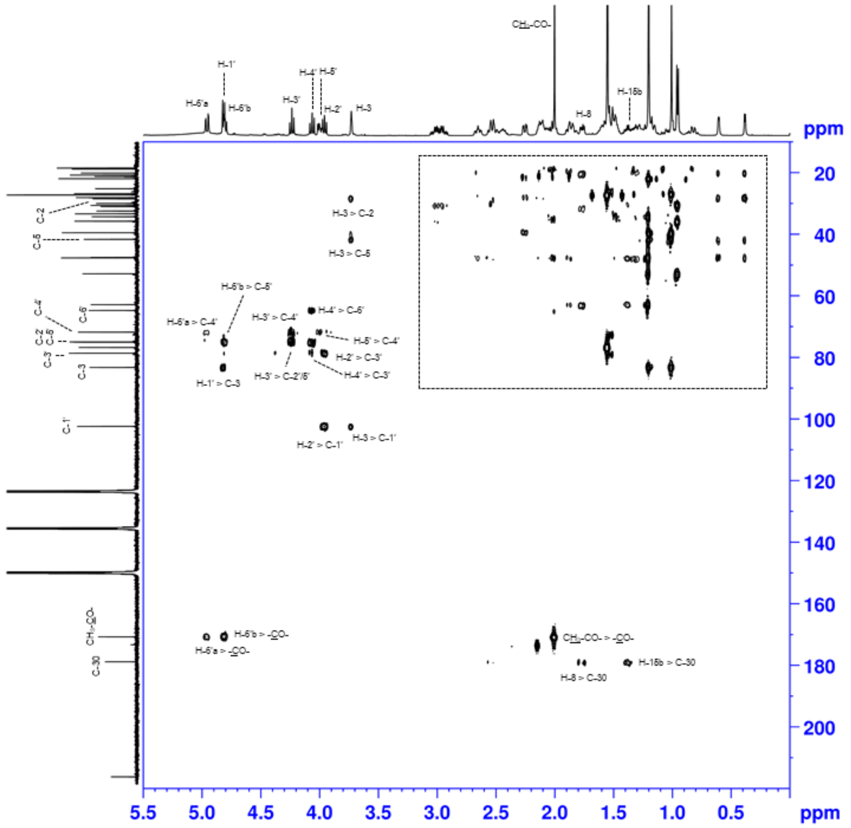

Figure S18. HMBC spectrum of **2**.

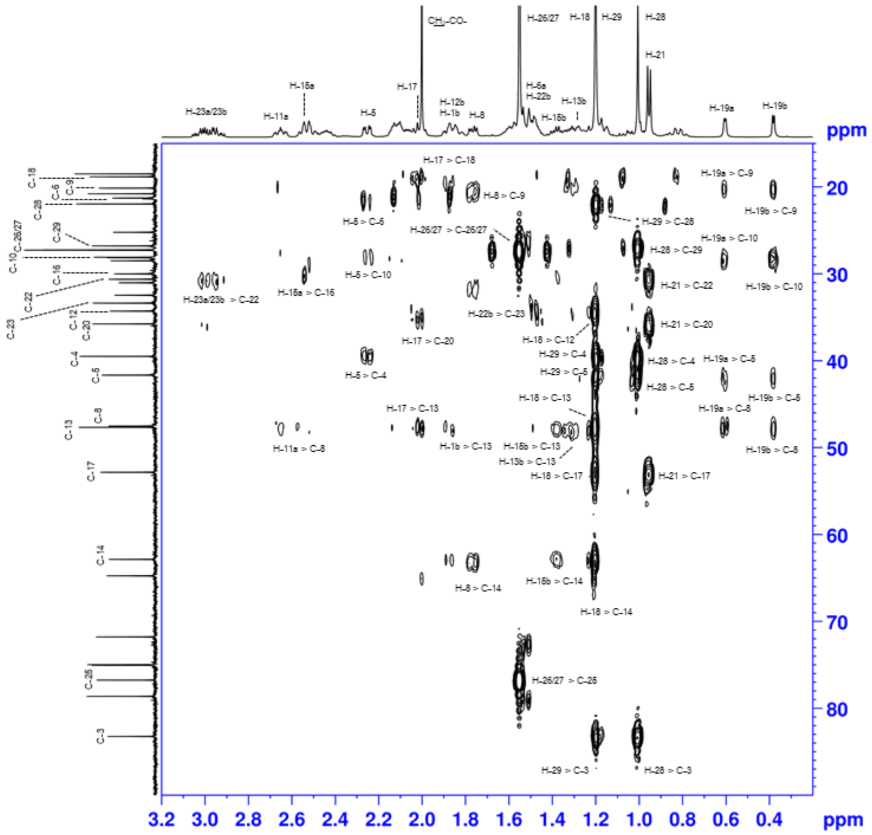

Figure S19. HMBC spectrum of **2** (enlarged figure).

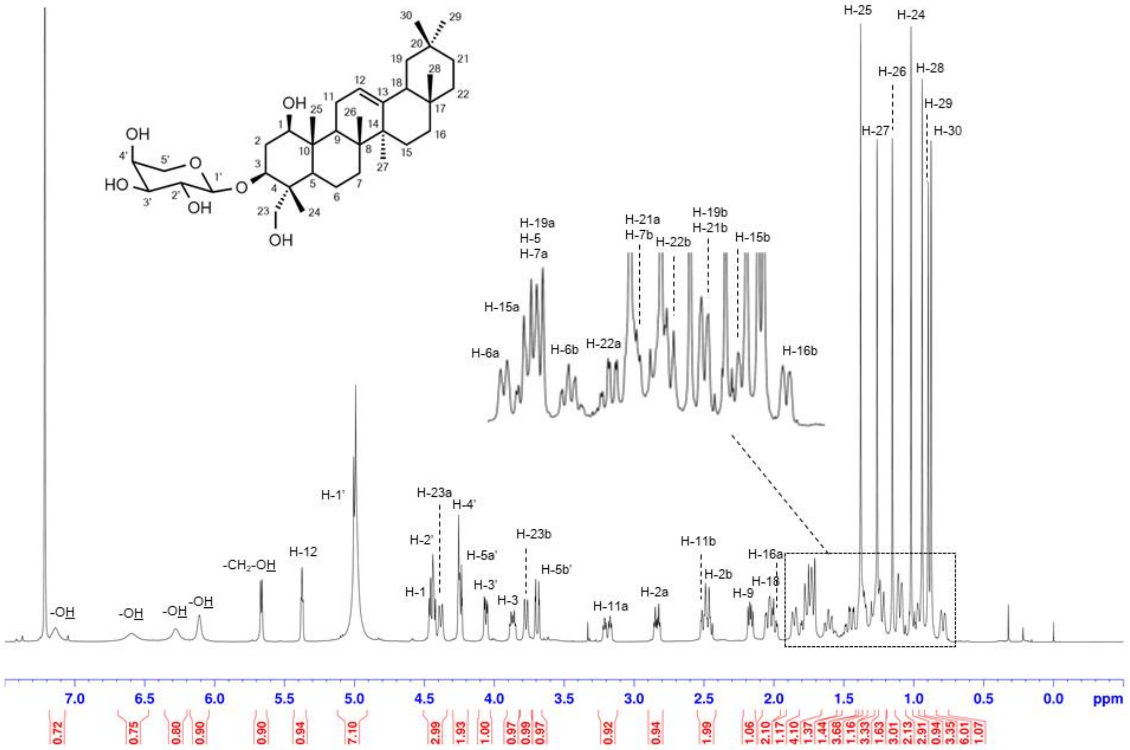

Figure S20. ^{1}H NMR spectrum of **3**.

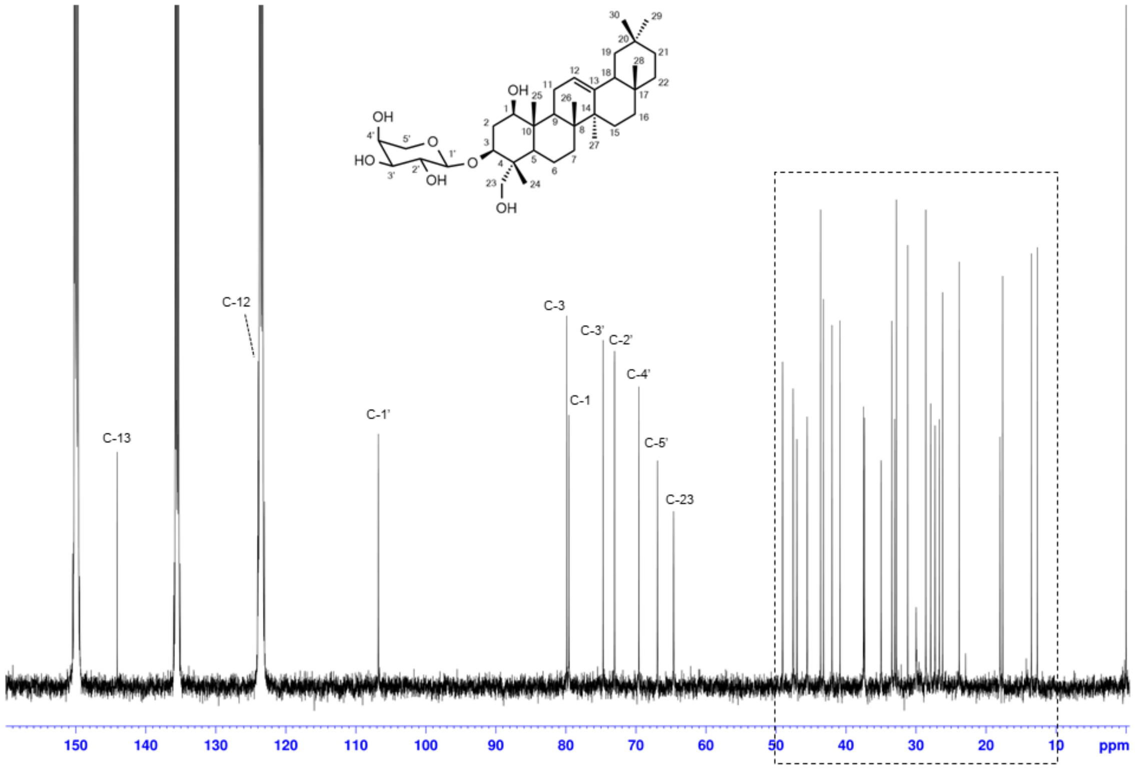

Figure S21. ^{13}C NMR spectrum of **3**.

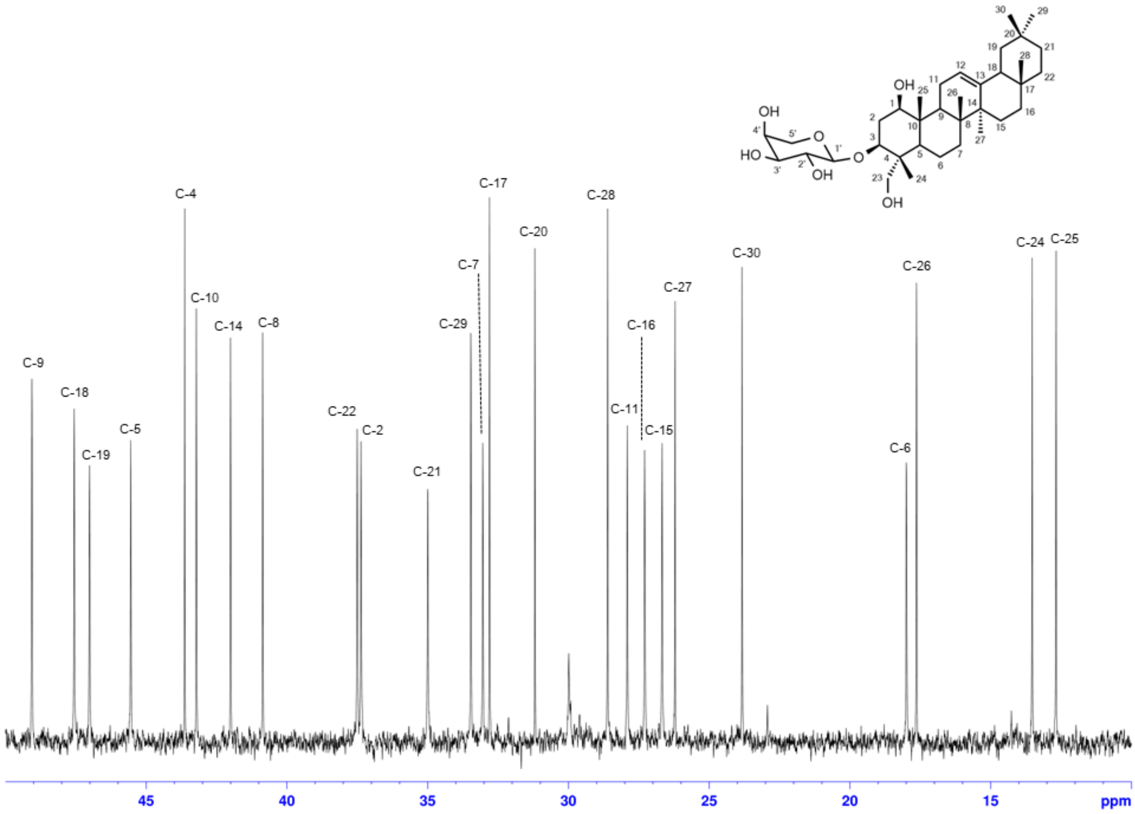

Figure S22. ^{13}C NMR spectrum of **3** (enlarged figure).

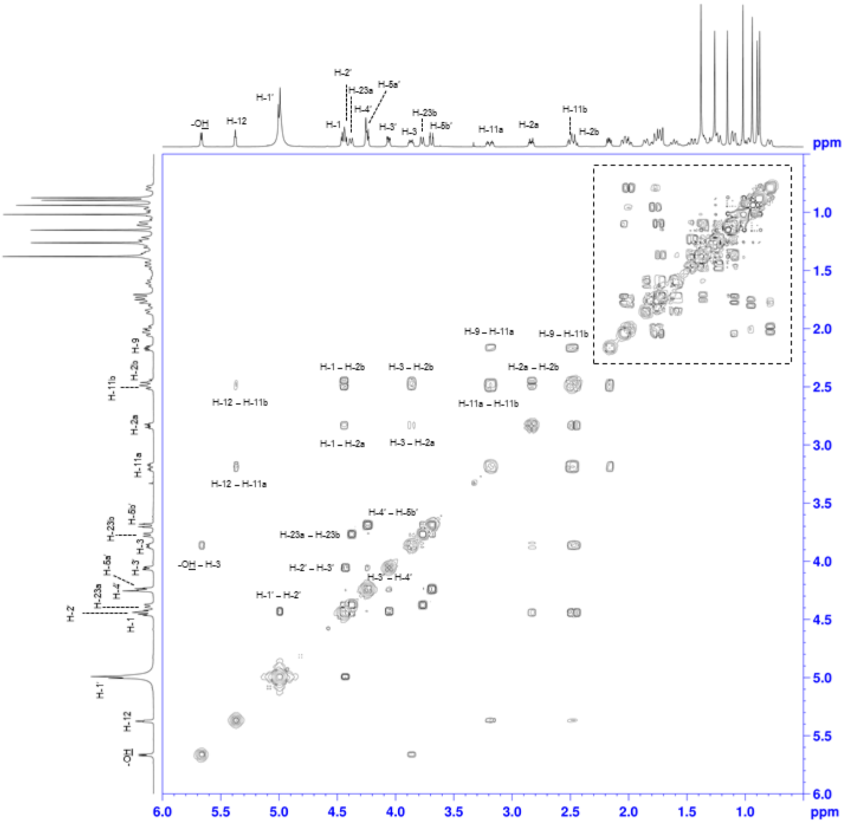

Figure S23. ^{1}H-^{1}H COSY spectrum of **3**.

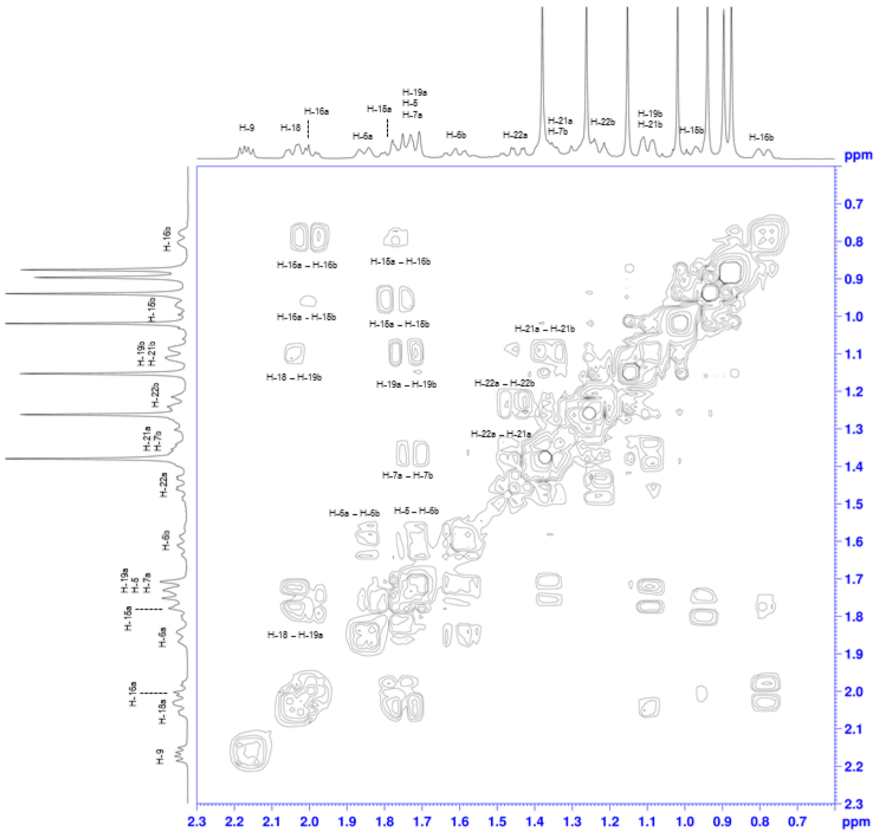

Figure S24. ^{1}H-^{1}H COSY spectrum of **3** (enlarged figure).

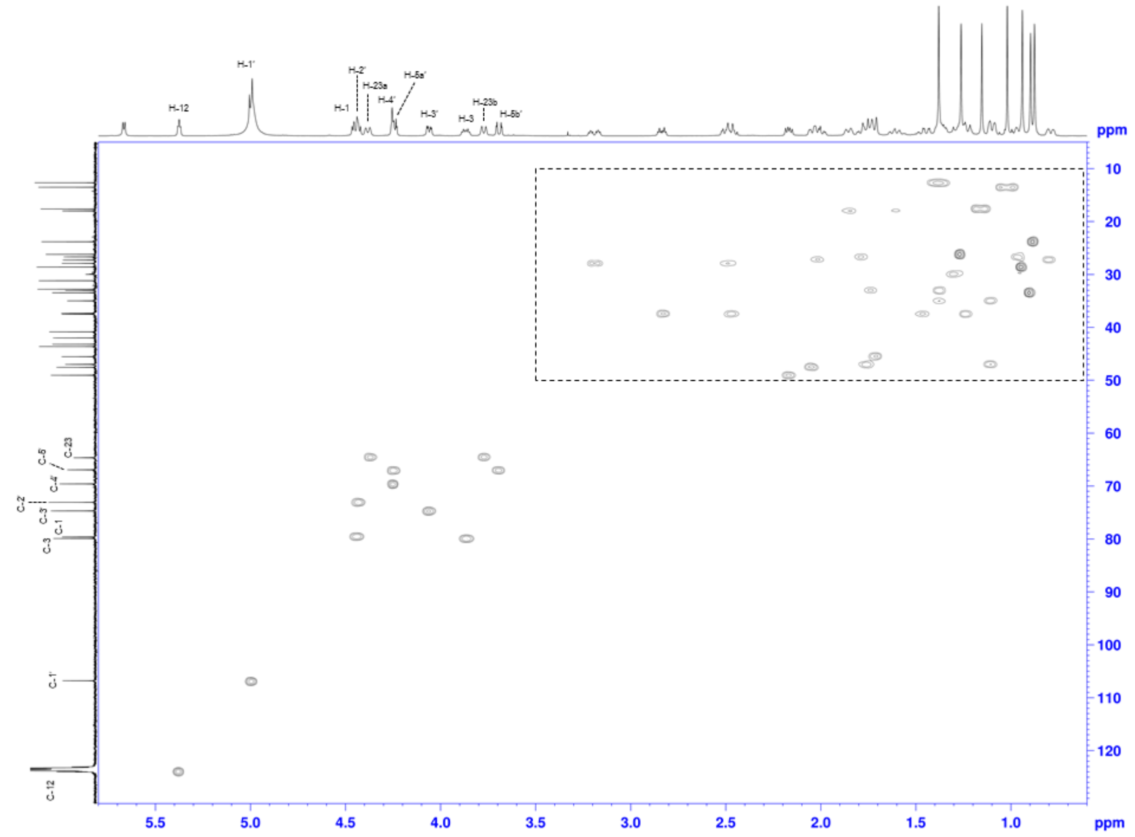

Figure S25. HSQC spectrum of **3**.

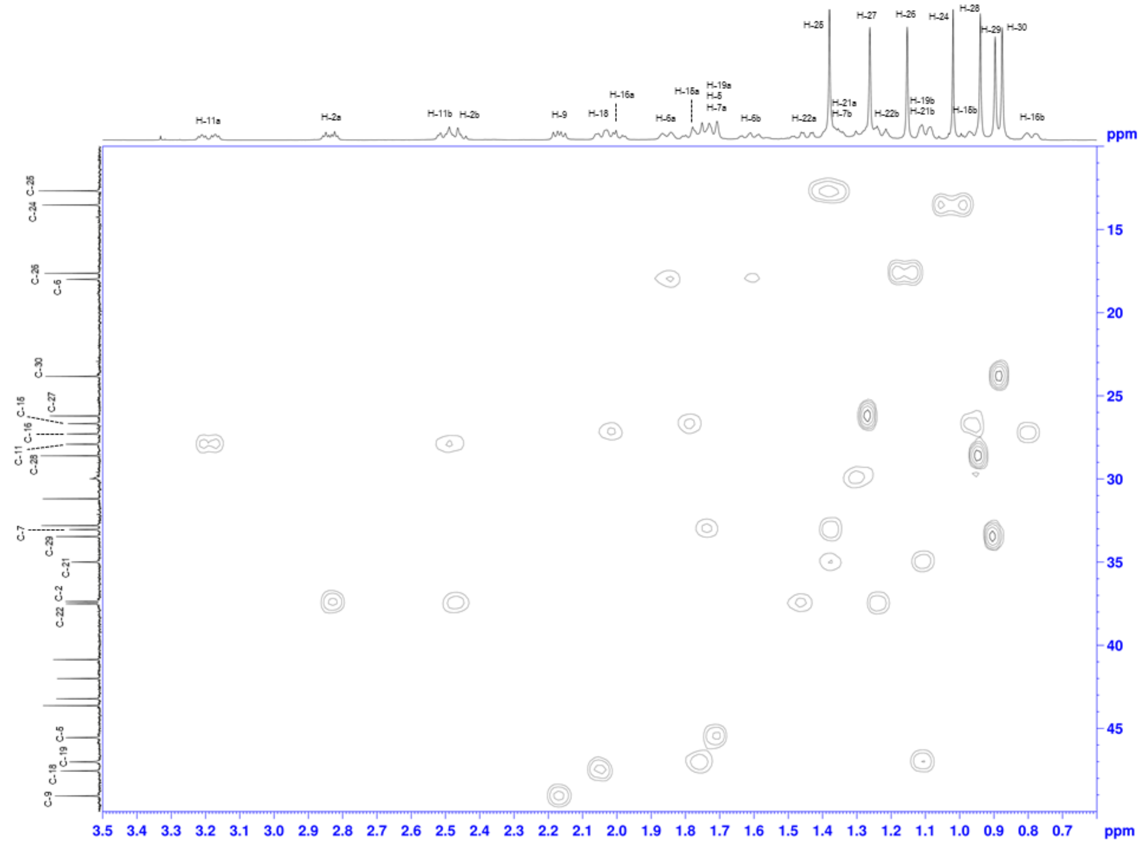

Figure S26. HSQC spectrum of **3** (enlarged figure).

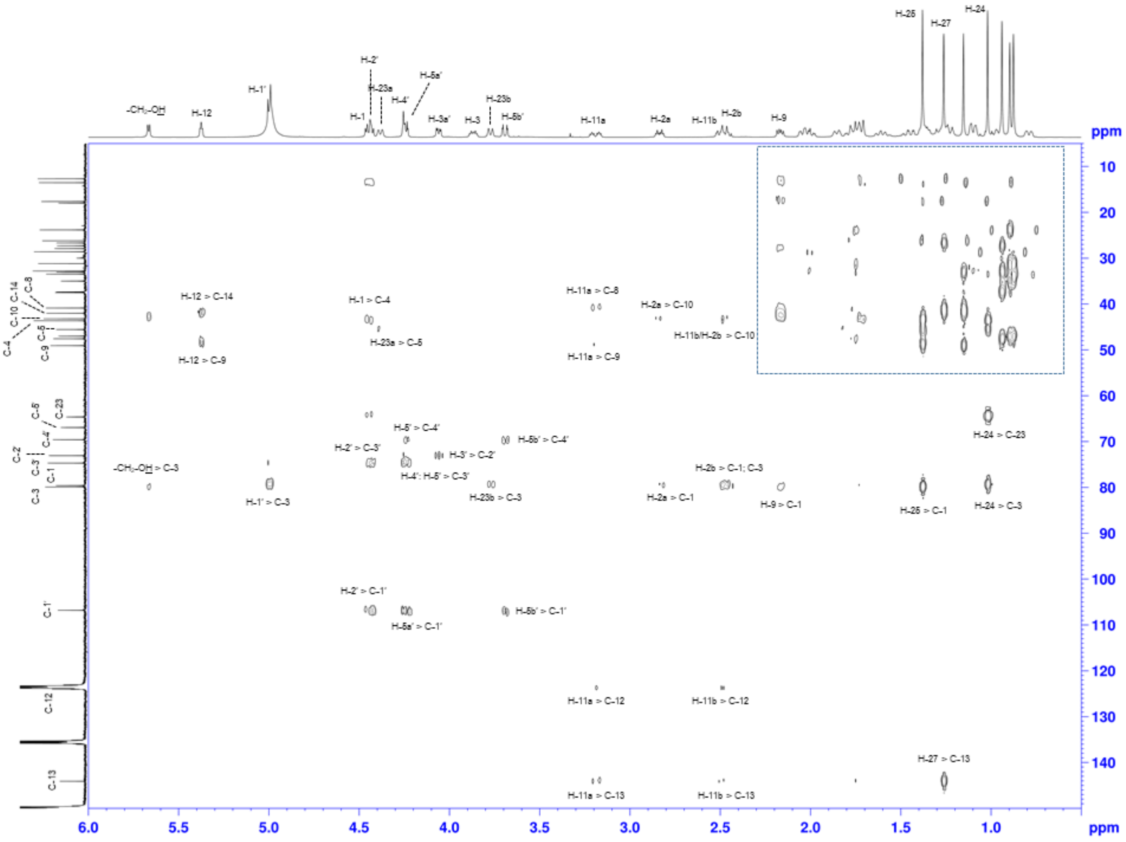

Figure S27. HMBC spectrum of **3**.

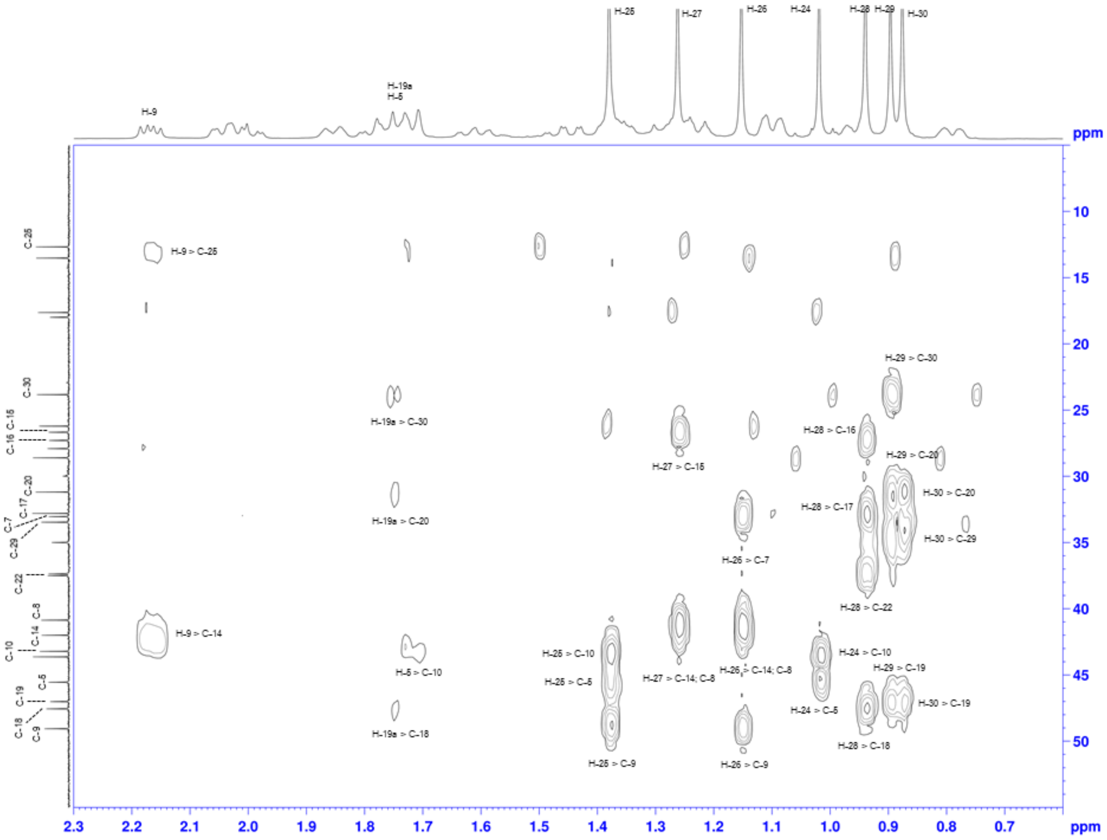

Figure S28. HMBC spectrum of **3** (enlarged figure).
